# Causation, not collinearity: Identifying sources of bias when modelling the evolution of brain size and other allometric traits

**DOI:** 10.1002/evl3.258

**Published:** 2021-11-09

**Authors:** Sam F. Walmsley, Michael B. Morrissey

**Affiliations:** ^1^ Scottish Oceans Institute, School of Biology, University of St. Andrews East Sands St. Andrews United Kingdom; ^2^ Dyers Brae House, School of Biology, University of St. Andrews Greenside Pl St. Andrews United Kingdom

**Keywords:** Allometry, brain size, causal inference, coevolution, comparative methods, correlated response to selection, reciprocal evolution

## Abstract

Many biological traits covary with body size, resulting in an allometric relationship. Identifying the evolutionary drivers of these traits is complicated by possible relationships between a candidate selective agent and body size itself, motivating the widespread use of multiple regression analysis. However, the possibility that multiple regression may generate misleading estimates when predictor variables are correlated has recently received much attention. Here, we argue that a primary source of such bias is the failure to account for the complex causal structures underlying brains, bodies, and agents. When brains and bodies are expected to evolve in a correlated manner over and above the effects of specific agents of selection, neither simple nor multiple regression will identify the true causal effect of an agent on brain size. This problem results from the inclusion of a predictor variable in a regression analysis that is (in part) a consequence of the response variable. We demonstrate these biases with examples and derive estimators to identify causal relationships when traits evolve as a function of an existing allometry. Model mis‐specification relative to plausible causal structures, not collinearity, requires further consideration as an important source of bias in comparative analyses.

## Impact Summary

Basic evolutionary theory suggests that some combinations of traits will evolve in response to one another. The most obvious example of this comes from allometric traits like brain size, which covary with body size. Given the tight correlation between brains and bodies, researchers interested in the evolution of brain size will use a body size‐corrected measure, or alternatively control for body size in a multiple regression framework. It has been suggested that controlling for body size in such models leads to inferential bias, stemming from underlying correlations or “collinearities” among traits (a widespread view in biostatistics). First, we clarify that collinearity does not result in spurious estimates and provides a roadmap for interpreting coefficients from models *with* and *without* body size. Second, we identify a separate source of bias complicating models of the diversification of correlated traits. Specifically, there are important causal implications rooted in the fact that, just as the selection on body size may cause a correlated response in brain size, selection on brain size may simultaneously cause a correlated response in body size. Standard methods (simple and multiple regression) for identifying selective agents driving the diversification of brain size cannot account for these causal processes, and fail to uncover unbiased estimates of the desired relationships in this setting. As a preliminary solution, we present a system of equations for estimating the direct effects of a given selective agent, accounting for reciprocal, correlated responses to selection. Biases stemming from mis‐specification of underlying causal pathways are underappreciated in evolutionary biology, and have significant implications for the development of comparative methods.

## Introduction

A principal aim of comparative biology is to identify selective agents that have driven the evolution of biological traits. One trait that has received considerable attention is brain size. Currently, myriad explanations for the evolution of brain size are supported by statistical models, creating a diversity of findings supporting different and often nonmutually exclusive hypotheses (Wartel et al. 2019). Like many other traits, brain size scales with body size. This is not surprising: larger bodies require a greater surface area of somatic nerve networks necessary for sensation, for example (Striedter 2005). As such, it has been common to use relative brain size (deviations from a fitted allometry) as a response variable when considering the “brainy‐ness” of a given individual or species. Preferably,^1^ body size is conditioned on or “controlled for” in a multivariate analysis such as multiple regression. However, whether absolute or body‐corrected brain size is the best predictor of cognitive ability remains unresolved, with statistical support for each view (Deaner et al. 2007), leading some researchers to test both measures in their analyses (Kverková et al. 2018).

However, the literature contains contradictory advice on the application of multiple regression in comparative analysis. A common argument is that collinearity (correlations among predictor variables) can lead to spurious findings in multiple regression analysis (Rogell et al. 2019). In the first part of this article, we provide an alternate view. Collinearity itself does not cause bias, despite the fact that the notion that it does is perpetuated by many biostatistics sources (see discussion in Morrissey and Ruxton 2018). In fact, the purpose of multiple regression is to separate direct effects from overall associations between predictor and response variables in the presence of associations among predictor variables. We discuss how, in the case of understanding drivers of the evolution of brain size, multiple regression is necessary and is exactly suited to control for correlated evolution of brain size resulting from the evolution of body size. However, in the second part of this article, we show that there are, nonetheless, scenarios where the particular form of statistical control for body size that multiple regression provides may lead to erroneous inference, insofar as associations between ecological drivers, brain size, and body size could arise from correlated responses of body size to adaptive evolution of brain size in response to ecological variables. These situations may be important, but are conceptually distinct to any supposed negative effects of collinearity on statistical inference. An understanding of biases that occur due to inclusion of consequences in multiple regression analyses, as though they were causes, while well understood in the causal inference literature, seems to be absent from the biostatistics literature, and could be very relevant beyond understanding brain and body size evolution. We discuss options for inference of direct effects of hypothesized selective agents on traits like brain size, that properly control for correlated effects arising due to the evolution of body size.

## Part 1: Addressing collinearity and multiple regression models in allometric studies

### THE NONPROBLEM OF SIGN REVERSALS

The argument that methods meant to control for body size can lead to inferential bias and spurious findings is widespread in the literature on brain size evolution (e.g., Mundry 2014; Gutierrez‐Ibanez et al. 2016), reflecting a broader concern about collinearity in the biostatistics literature (e.g., Zuur et al. 2009). It was recently suggested that one source of such bias is sign reversals for the effect of selective agents on brain size (Rogell et al. 2019).

We argue that such sign reversals are, however, unproblematic. Because simple and multiple regression are tools designed to answer different biological questions, their numerical outputs need not coincide.

Continuing with the example of the diversification of brain size, simple regression answers the question, “is there an overall relationship between the selective agent and brain size?” In contrast, multiple regression estimates “direct” effects of each predictor on the response variable when all other predictors are held constant. Thus for our example, multiple regression of brain size on a putative selective agent and body size answers the question: “is brain size associated with the selective agent beyond the degree expected if it were evolving in response to the evolutionary allometry, given that the agent and body size may be correlated?” In this section, we proceed with the brain–body example of an allometric trait, but the reasoning applies to any allometric trait, and to how simple and multiple regression analyses should be understood in general. Morrissey and Ruxton (2018) provide more detailed and general elaboration on the points that we describe here in the context of the more specific case of brain size and body size.

We now present biological interpretations of two scenarios where results of simple and multiple regression may superficially appear to conflict. First, imagine that a selective agent causes an increase in body size, and body size, in turn, causes an increase in brain size. Here, simple regression should recover a positive agent‐brain size relationship while multiple regression should estimate an effect of the agent on brain size near 0 when the body size is included. That simple and multiple regression both correctly recover these properties of such a system is demonstrated in the supplement in numerical example 1. These different numerical results are expected and are biologically correct, if the questions to which they provide answers are clearly kept in mind. The simple regression confirms that an overall association exists between the agent and brain size. The multiple regression confirms that there is no association of the agent with brain size, over and above, the expected allometry.

Second, imagine that (large values of) a selective agent cause decreases in brain size, but large increases in body size, and that evolution of large body size, in and of itself, causes increases in brain size. Depending on the magnitudes of each effect, it is possible that the positive contribution of the indirect effect of the selective agent on brain size, via body size, could overwhelm the negative direct effect, leading to a positive overall association (supplemental materials numerical example 2 uses values that generate this pattern). This positive overall association would manifest as a positive slope in a simple regression of brain size on the selective agent. However, in this scenario, a multiple regression analysis would be able to discern that, independently of body size and the resulting overall positive association, the selective agent had a negative direct effect on brain size. This would be an example of a sign reversal, though the different signs of the simple and multiple regression coefficients would not indicate any erroneous inference. Rather, they would be correct representations of two complementary parts of the biological system.

More generally, collinearity is not a source of bias, though it is a prerequisite for sign reversals, or indeed for a difference between simple and partial regression coefficients to occur. With noncollinear variables, the numerical values of simple and partial regression coefficients coincide. Granted, teasing apart the direct effects of increasingly collinear predictors is statistically challenging, and ultimately impossible if predictors are identical (or perfectly correlated). Thus, direct effects of increasingly collinear predictors will be less precise, or alternatively, require a larger dataset to achieve a precise estimate. Nevertheless, the fact that collinearity brings the distinction between simple and multiple regression into focus does not imply that the distinction was not already present, nor that the results are inconclusive. Oppositely, these differences are proof of the usefulness of multiple regression as the appropriate tool to answer specific questions about relationships among biological variables.

### CONDITIONING ON BODY SIZE CAN BE NECESSARY TO CONTROL FOR SOME FORMS OF NONCAUSAL ASSOCIATION

The key issue, then, is whether the questions one wants to ask correspond to the answers provided by simple regression, multiple regression, or both. In other words, does the biology suggest that one should control for some correlated variable (e.g., body size), using multiple regression, and thus, generate an estimate which is apt to be different from the overall association between some focal predictor (e.g., a selective agent) and the response (e.g., brain size)? The standard reason to control for a correlated trait (or measuring body‐corrected brain size, for example) is the desire to estimate the independent effects of each predictor on the response, agnostic to a variety of (possibly unobserved) possible causes driving the association. However, we argue that recasting the decision to control for a correlated trait (e.g., body size) in terms of specific causal processes would be useful for determining what analysis is necessary for any given biological question.

First, we briefly introduce the process of making causal inferences. While scientists are often interested in causal relationships, statistical models alone can only provide evidence of association. However, judicious application of assumptions about possible causal relationships can yield substantial opportunities to make causal inferences from associations, even for complex observational data (Wright 1920; Pearl 2000). In brief, the aim of causal inference is to (1) establish a plausible network of causation among a set of variables, and (2) block paths of association separate from the key causal relationship of interest (see Pearl et al. 2016). These “backdoor” paths are noncausal links between two variables that traverse against the direction of causal arrows and which can be blocked by statistical control. Once these paths are blocked, and assuming that the causal network has been correctly specified, the remaining statistical associations can be interpreted as causal effects. Thus, whether a variable should be conditioned on (i.e., included as a covariate in a multiple regression model) depends on the underlying causal structure, a feature related to, but not identical with, the associations or collinearities between them.

**Figure 1:**
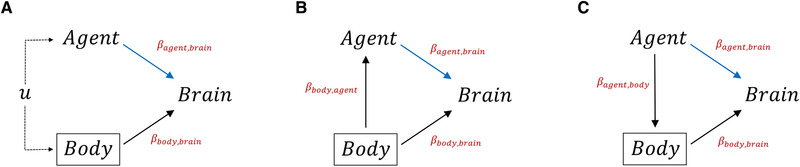
Directed acyclic diagrams showing possible causal relationships where controlling for body size is necessary to identify the direct effect of the agent on brain size. (A) The selective agent and body size are both influenced by unmeasured variables u. (B) Body size is a common cause of a selective agent and brain size, confounding the simple regression of brain size on the agent. (C) The selective agent causes diversification in body size which results in a correlated response in brain size. Here, controlling for body size via multiple regression, blind to the possible causal pathway(s) at play, will reveal the direct causal effect of the agent on brain size.

The scenarios illustrated in Figure 1 all depict causal models under which the multiple regression of brain size on body size and a selective agent will return correct estimates of the direct effects of both predictor variables on brain size. Whatever the nature of the association of the agent and body size, be it some unmeasured common cause, an effect of the agent on body size, or an effect of body size on the agent, multiple regression will serve to estimate the effects of both variables on brain size.

An important caution is that the effects estimated by multiple regression are direct effects, and these need not necessarily represent the total causal effects of a variable on a response, if mediating variables are present. In Figure 1C, the agent causes brain size to evolve via two paths, a direct path, and a path that is mediated by body size. If total effects are of interest, then additional assumptions about the causal structure of associations among predictor variables are necessary. Insofar as it may be reasonable to hypothesize the specific causal structure in Figure 1C, the simple regression of brain size on the selective agent reveals the total effect via both pathways, while multiple regression reveals the direct effect. In more general path models, simple regression will not typically estimate total effects (i.e., including direct and mediated paths), and recourse to formal path analysis (Wright 1934; Shipley 2000) will be necessary.

### PART 1: KEY POINTS

When one wants to distinguish the contributions of direct effects of a selective agent on brain size from on the one hand, and associations arising because, on the other hand, brain size may evolve as a consequence of body size, when it is body size that has diversified in response to the selective agent, we can use multiple regression to make unbiased estimates of the relevant quantities. In fact, in such a scenario it is *necessary* to use multiple regression to make estimates of such direct effects. That the simple and partial regression coefficients may differ from the overall association of selective agents with brain size need not be seen as any indication that control for body size has generated any inferential bias. A slightly more formal recourse to statistical theory of regression may bring a perspective that sums up the issue nicely: Partial regression coefficients, obtained in the standard way using Ordinary Least Squares, are Minimum Variance Unbiased estimators (MVUB) of (linear) effects (Rao 1973). The MVUB property means that not only are partial regression unbiased by collinearity, but it is mathematically provable that, among unbiased estimators, a more precise estimator cannot be obtained.

## Part 2: Addressing the possibility that body size could evolve as an indirect effect of adaptive diversification of brain size

### A SECOND PATHWAY OF CORRELATED RESPONSES TO SELECTION

**Figure 2:**
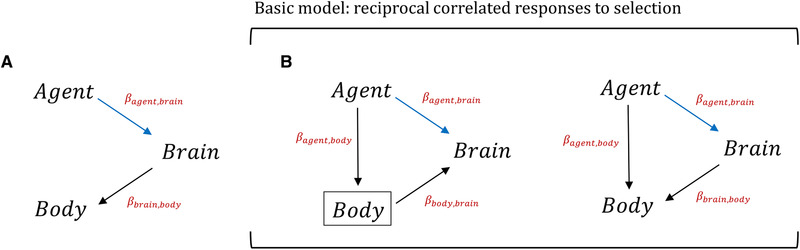
Directed acyclic diagrams showing possible causal relationships where controlling for body size will not recover the direct effect of the agent on brain size. (A) The selective agent causes diversifying selection in brain size which results in a correlated response in body size. Here, conditioning on body size will return an underestimate of the desired effect. **(B)** Reciprocal correlated responses to selection resulting from direct selection on body size (left; same as Fig. 1B) and brain size (right). Evolutionary theory suggests, as the most basic model, that both pathways in (**B)** may be occurring simultaneously, meaning that neither simple nor multiple regression can identify a direct causal effect of a selective agent on brain size.

The above situation (Part 1) is likely the more familiar pattern, where including an additional variable helps to distinguish a desired direct effect from an overall association. However, adding variables to a multiple regression can also *interfere* with one's ability to make causal inferences. In the context of evolutionary diversification of correlated traits (e.g., brain size and body size), it may be necessary to consider an additional pathway of correlated responses: just as the selection on a correlated trait may induce a response in the focal trait, selection on the focal trait may lead to diversification of the correlated trait (Fig. 2A; the behavior of multiple regression analysis under this scenario is demonstrated by numerical example 3 in the Supporting information). For example, imagine that a selective agent, for example, habitat complexity, causes diversifying selection in brain size, with a correlated response in body size. Here, body size would be associated with habitat complexity solely via a correlated response to selection. Inasmuch as body size may evolve as a correlated response to habitat‐driven evolution of brain size, including body size as a predictor will, to some extent, amount to including a consequence of brain size evolution as though it was a cause. A more formal discussion of this “case‐control” or “selection” bias problem can be found in the causal inference literature (Bareinboim and Pearl 2012; Pearl 2013; Cinelli et al. 2020).

As far as the causal structure of direct and correlated selection is concerned, the possibility of both brain size and body size evolving via direct and correlated selection amounts to both diagrams in Figure 2B occurring simultaneously. Controlling for body size in this case would have two consequences: (1) to whatever extent brain size may be a correlated response to the evolution of body size, inclusion of body size as a predictor variable is essential to estimate the direct effect of the agent on brain size, but (2) to whatever extent body size may be a correlated response to evolution of brain size, its inclusion in the regression model will generate bias. In the language of causal inference, where body size may be a common *outcome* of brain size and the selective agent (Fig. 2B right), it is considered a “collider” variable, on which should generally not condition (Pearl 2013). A toy example where both traits may be evolving both due to direct and indirect pathways is provided in the Supporting information in numerical example 4.

### UNBIASED ESTIMATION OF DIRECT EFFECTS OF SELECTIVE AGENTS UNDER DIRECT AND CORRELATIONAL RESPONSES TO SELECTION

In principle, if a single one of the described causal diagrams could be confidently assumed for a selective agent, body size, and brain size in a given study, the consequences of conditioning on body size should be clear. In such cases, it would be relatively simple to calculate unbiased estimates of the direct effect of the agent on brain size. However, the null expectation from microevolutionary theory suggests that brain size and body size may be evolving as the result of both direct and correlated responses to selection (Lande 1979; Walsh and Lynch 2018) – at the very least it would be imprudent to rule out these causal pathways. As such, the basic statistical model for the diversification of allometric traits needs to accommodate for reciprocal, simultaneous direct and correlated responses to selection. As conditioning on body size is necessary to identify the causal effect under one pathway (Fig. 2B left) but will lead to bias in the other (Fig. 2B right), neither simple nor multiple regression will recover an accurate direct effect when both pathways may occur simultaneously.

As a preliminary solution to this problem, we present a system of equations whose solution yields method of moments (MoM) estimators that recover the desired, direct effects of a selective agent on a (possibly) diversifying trait, like brain size, allowing for correlated responses to selection. Let $\beta_{a,br}$ and $\beta_{a,bo}$ be the direct effects of a hypothesized selective agent on brain size and body size, respectively. Let $\Sigma_{0}$ be the variance–covariance matrix of brain size and body size among taxa that would exist without the influence of the selective agent,

$$\;\Sigma_{0}=\left[\begin{array}{cc} \sigma_{bo,0}^{2} & \sigma_{br,bo,0}\\ \sigma_{br,bo,0} & \sigma_{br,0}^{2} \end{array}\right]\;.$$

The allometric regression of brain size on body size, in the absence of (or controlling for) the action of the hypothesized diversifying agent, is

(1) $$\beta_{bo,br,0}=\;\frac{\sigma_{br,bo,0}}{\sigma_{bo,0}^{2}},$$

and the corresponding regression of body size on brain size, conditional on the hypothesized agent, is

(2) $$\beta_{br,bo,0}=\;\frac{\sigma_{br,bo,0}}{\sigma_{br,0}^{2}}.$$

From the rules of path analysis (linear transformations of random variables), the part of the covariance of the selective agent and brain size arising from the direct effect of the selective agent on brain size is $\sigma_{a}^{2}\cdot \beta_{a,br}$. The corresponding part of the covariance of the selective agent and body size, arising from the direct diversifying effect only, is $\sigma_{a}^{2}\cdot \beta_{a,bo}$.

If the correlated diversification of brain size in response to the selective agent acting directly on body size acts according the evolutionary allometry that would be obtained in the absence of the selective agent, that is, according the regression in Equation (1), then correlated diversification in brain size, as a result of direct diversifying selection on body size, would contribute a $\sigma_{a}^{2}\cdot \beta_{a,bo}\cdot \beta_{bo,br,0}$ to the covariance of the diversifying agent and brain size (Lande 1979; Walsh and Lynch 2018). This pattern of diversification is consistent with a simple quantitative genetic model or responses of correlated traits (Walsh and Lynch 2018), that would arise if evolutionary allometries resulted from genetic correlations among traits. However, this model also reflects the wider array of biological processes that may generate evolutionary allometries, for example, as would arise if populations tracked selective optima for brain size and body size where these optima were correlated for purely ecological reasons. Similar to the allometric evolution of brain size, the indirect contribution to the covariance of the agent and body size, resulting from direct diversifying selection on brain size, is $\sigma_{a}^{2}\cdot \beta_{a,br}\cdot \beta_{br,bo,0}$.

The variance in brain size caused by direct and correlated diversifying selection according to the hypothesized agent is given by $\sigma_{a}^{2}\cdot {\left(\beta_{a,br}+\beta_{a,bo}\cdot \beta_{bo,br,0}\right)}^{2}$, and the contribution of the agent to variance of body size is $\sigma_{a}^{2}\cdot {\left(\beta_{a,bo}+\beta_{a,br}\cdot \beta_{br,bo,0}\right)}^{2}$. The covariance of brain and body size induced by the selective agent is $\sigma_{a}^{2}\cdot \left(\beta_{a,\mathrm{br}}+\beta_{a,\mathrm{bo}}\cdot \beta_{\mathrm{bo},\mathrm{br},0}\right)\left(\beta_{a,\mathrm{bo}}+\beta_{a,\mathrm{br}}\cdot \beta_{\mathrm{br},\mathrm{bo},0}\right)$.

It is now possible to write down the variances and covariances among the selective agent, brain size, and body size, in terms of $\beta_{a,br}$, $\beta_{a,bo},$ and $\Sigma_{0}$ (n.b., the variance of the selective agent, $\sigma_{a}^{2}$ is taken to be given),

(3a) $$\sigma_{br}^{2}=\;\sigma_{br,0\;}^{2}+\sigma_{a}^{2}\cdot \;{\left(\beta_{a,br}+\beta_{a,bo}\cdot \beta_{bo,br,0}\right)}^{2},$$

(3b) $$\sigma_{bo}^{2}=\;\sigma_{bo,0\;}^{2}+\sigma_{a}^{2}\cdot \;{\left(\beta_{a,bo}+\beta_{a,br}\cdot \beta_{br,bo,0}\right)}^{2},$$

(3c) $$\begin{array}{ccc} \sigma_{br,bo} & = & \;\sigma_{br,bo,0\;}^{2}+\sigma_{a}^{2}{\left(\beta_{a,br}+\beta_{a,bo}\cdot \beta_{bo,br,0}\right)}^{2}\\ & & \times \;{\left(\beta_{a,bo}+\beta_{a,br}\cdot \beta_{br,bo,0}\right)}^{2}, \end{array}$$

(3d) $$\sigma_{a,br}=\;\sigma_{a\;}^{2}\left(\beta_{a,br}+\;\beta_{a,bo}\cdot \beta_{bo,br,0}\right),$$

(3e) $$\sigma_{a,bo}=\;\sigma_{a\;}^{2}\left(\beta_{a,bo}+\;\beta_{a,br}\cdot \beta_{br,bo,0}\right).$$

The solution to this system of equations (by substituting the definitions of $\beta_{bo,br_{0}}$, $\beta_{br,bo_{0}}$ in terms of $\Sigma_{0}$ given in Equations (1) and (2) with algebraic simplification) gives MoM estimators for $\beta_{a,br}$, $\beta_{a,bo}$

(4a) $$\begin{array}{ccc} & & \beta_{a,\mathrm{br}}=-\;\frac{\left(\sigma_{a,\mathrm{br}}^{2}-\;\sigma_{a\;}^{2}\sigma_{\mathrm{br}\;}^{2}\right)\left(\sigma_{a,\mathrm{bo}}\sigma_{\mathrm{br},\mathrm{bo}}-\;\sigma_{a,\mathrm{br}}\sigma_{\mathrm{bo}}^{2}\right)}{\sigma_{a}^{2}\left(\sigma_{a,\mathrm{bo}}^{2}\sigma_{\mathrm{br}}^{2}-2\sigma_{a,\mathrm{bo}}\sigma_{a,\mathrm{br}}\sigma_{\mathrm{br},\mathrm{bo}}+\;\sigma_{a,\mathrm{br}}^{2}\sigma_{\mathrm{bo}}^{2}+\sigma_{\mathrm{br},\mathrm{bo}}^{2}\sigma_{a}^{2}-\sigma_{a}^{2}\sigma_{\mathrm{bo}}^{2}\sigma_{\mathrm{br}}^{2}\right)}, \end{array}$$

(4b) $$\begin{array}{ccc} & & \beta_{a,\mathrm{bo}}=-\;\frac{\left(\sigma_{a,\mathrm{bo}}^{2}-\;\sigma_{a\;}^{2}\sigma_{\mathrm{bo}\;}^{2}\right)\left(\sigma_{a,\mathrm{bo}}\sigma_{\mathrm{br}}^{2}-\;\sigma_{a,\mathrm{br}}\sigma_{\mathrm{br},\mathrm{bo}}\right)}{\sigma_{a}^{2}\left(2\sigma_{a,\mathrm{bo}}\sigma_{a,\mathrm{br}}\sigma_{\mathrm{br},\mathrm{bo}}-\sigma_{a,\mathrm{bo}}^{2}\sigma_{\mathrm{br}}^{2}-\;\sigma_{a,\mathrm{br}}^{2}\sigma_{\mathrm{bo}}^{2}-\sigma_{\mathrm{br},\mathrm{bo}}^{2}\sigma_{a}^{2}+\sigma_{a}^{2}\sigma_{\mathrm{bo}}^{2}\sigma_{\mathrm{br}}^{2}\right)}. \end{array}$$

While cumbersome, these expressions show that it is possible, in the presence of reciprocal correlated responses to selection, to identify the direct effects of the agent on both traits. This inference is possible using only observable quantities (i.e., variances and covariances of the selective agent, brain size, and body size), under the standard model of direct and correlated responses to selection from evolutionary quantitative genetics. The application of these estimators in a scenario where multiple regression fails is given in numerical example 4 in Supporting Information.

**Figure 3:**
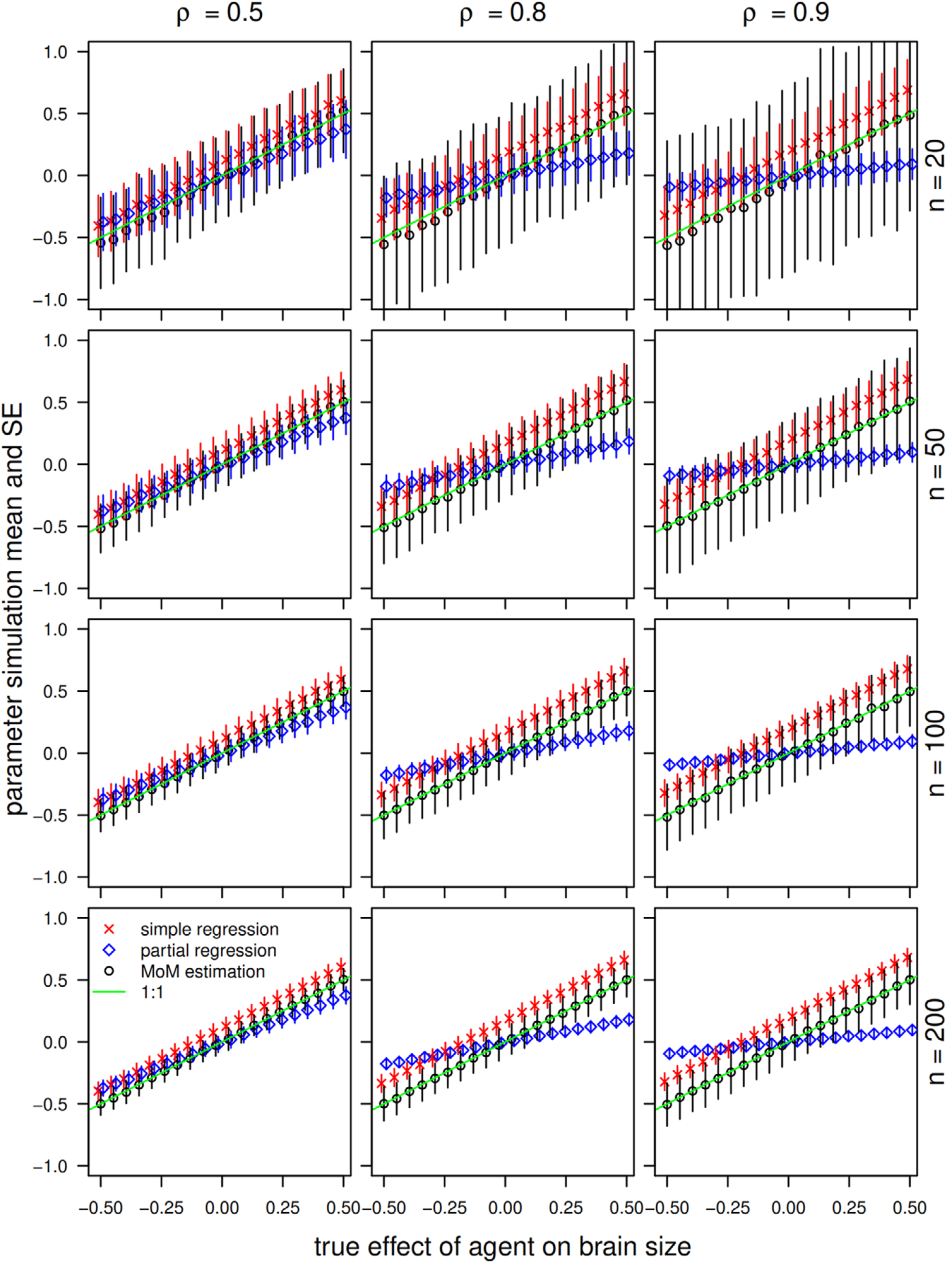
Simulations comparing the results of simple regression, partial regression, and our estimator when both brains and bodies diversify as a result of direct and correlated responses to selection. For this causal scenario, the true effect cannot be estimated by either including or excluding the correlated trait (body size) from a regression model.

We explored the behavior of these estimators (Eq. 4a/b) by simulating agent, brain size, and body size data across a range of sample sizes (20, 50, 100, 200), background allometric correlations (0.5, 0.8, 0.9), nonmediated causal effects of the agent on brain size (−0.5 to 0.5), and a constant nonmediated causal effect of the agent on body size (0.2). We find that the approach provides unbiased estimates of direct effects on diversification when there are reciprocal correlated responses to selection, whereas neither simple nor multiple regression recover the correct estimate, in the important general case when both brain and body size can evolve in response to direct and correlated selection (Fig. 3). As in multiple regression, statistical uncertainties in estimates are very large when correlations among predictor variables are high. For traits with strong allometries (e.g., allometric correlations on the order of 0.9), much larger sample sizes than are typically used in comparative studies may be required for unbiased estimation of effects of hypothesized agents of evolutionary diversification on focal traits.

In the simulations depicted in Figure 3, values of simple regressions, partial regressions, and effects in systems with reciprocal indirect evolutionary effects rarely coincide. We have only simulated a modest range of parameter values, with the intention of illustrating the basic principle, and to give an initial indication of the kinds of sample sizes required (and especially how desirable values of n might depend on the degree of collinearity). A more comprehensive range of simulation parameters would generate additional outcomes. For example, in Figure 3, all simple regressions of brain size on the agent exceed the corresponding direct effects. This is because all allometries are positive, and we considered only one (positive) example value of the effect of the agent on body size. If the effect of the agent on brain size was negative, for example, then simple regressions for brain size would be less (more negative) than the direct effects. The scenario for negative agent‐body size effects is presented as an additional figure in the Supporting information, and all simulation code is given so that further ranges of parameter values may easily be considered.

The solution to the system of equations in Equation (3) also yields estimators of the variances and covariance of the brain size and body size that would obtain in the absence of the selective agent. These (symbols as defined above) are $\sigma_{br,0}^{2}=\;\sigma_{br}^{2}-\frac{\sigma_{a,br}^{2}}{\sigma_{a}^{2}}$, $\sigma_{bo,0}^{2}=\;\sigma_{bo}^{2}-\frac{\sigma_{a,bo}^{2}}{\sigma_{a}^{2}}$ and $\sigma_{br,bo,0}=\;\sigma_{br,bo}-\frac{\sigma_{a,bo}\sigma_{a,br}}{\sigma_{a}^{2}}$. These quantities are not currently part of the repertoire of parameters estimated by evolutionary biologists. However, they could become useful, for example, for disentangling the contributions of developmental associations versus adaptive evolution in shaping evolutionary allometries. The allometric relation (regression of brain size on body size) defined by these variances and covariances is, thus, $\beta_{br,bo,0}=\;\frac{\sigma_{br,bo,0}}{\sigma_{bo,0}^{2}}\;=\frac{\sigma_{br,bo}-\frac{\sigma_{a,bo}\sigma_{a,br}}{\sigma_{a}^{2}}.\;}{\sigma_{bo}^{2}-\frac{\sigma_{a,bo}^{2}}{\sigma_{a}^{2}}}\;$ in terms of observable quantities. This particular allometry is interesting. It might be thought of as the average static genetic allometry that would be consistent with the evolutionary allometry and the apparent effects of the selective agent on brain size and body size. This allometry need not be equal to the actual evolutionary allometry, and indeed any difference between this static allometry and the evolutionary allometry might be interpretable as the contribution of the selective agent to the actual evolutionary allometry, relative to the evolutionary allometry that would be obtained in the absence of adaptation in response to the selective agent.

### THE TREATMENT OF PREDICTOR AND RESPONSE VARIABLES IN REGRESSION MODELS

Before applying this method to the datasets used in Rogell et al. (2019), we first clarify a point pertaining to the calculation of partial regression coefficients. A seemingly helpful approach is to report the partial regression coefficients for effects of selective agents on brain and body sizes using regressions of the selective agent (as the response variable) on brain size and body size as in Rogell et al 2019. This appears convenient in that it provides coefficients relating the selective agent to both traits, from a single model. However, it should not be used to address the question of effects of selective agents on traits (potentially controlling for other traits). While, under certain standardizations, simple regressions of *y* on *x* and *x* on *y* take the same numerical values, the same is not true of multiple regression (numerical example 5 in Supporting information provides a practical demonstration). Furthermore, the regression of a selective agent on two highly correlated traits (in this case, brain size and body size) leads to a situation where the variables that are treated as predictors are highly correlated, and thus, potentially greatly exaggerates any perceived problems that arise because of collinearity. Under separate multiple regressions of each trait on the agent, while controlling for the other trait, the high collinearity between traits that tends to occur in studies of brain size and body size does not emerge, because brain size and body size are not both the predictor variables. Partial regression coefficient estimates for effects of selective agents on brain size and body size using regressions of the selective agent on brain size and body size, thus, do not necessarily bear on the quantities regularly calculated and reported by biologists conducting comparative studies using multiple regression analysis. Though the more important aspect of the present article is to clarify that divergence between coefficients from simple and multiple regression does not indicate that one or the other is biased, and so this issue with the specific partial regression coefficients used is not critical, we do provide versions of plots found in Rogell et al. (2019), with correctly calculated partial regression coefficient estimates, in Supporting information.

### DIRECT EFFECTS IN THE BRAIN–BODY ALLOMETRY EXAMPLES, ALLOWING FOR CORRELATED EVOLUTION OF BOTH TRAITS

**Figure 4:**
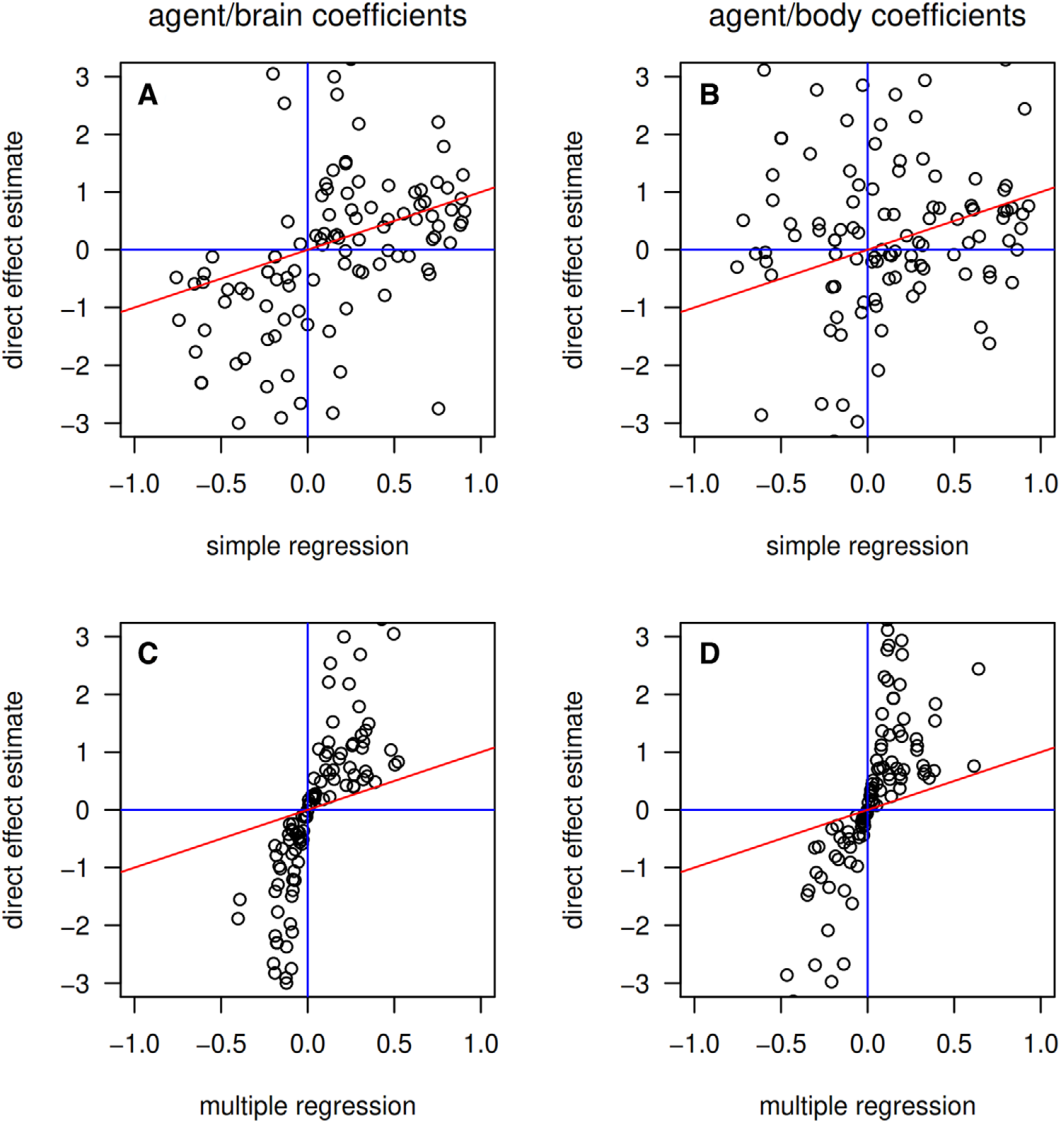
Comparisons among estimators of relationships between brain size, body size, and putative agents of selection. Estimates of direct effects that account for a basic model of reciprocal correlated evolution are compared to simple regression coefficients (A,B), and multiple regression coefficients (C,D), for agent‐brain size relationships (A,C), and agent‐body size relationships (B,D).

We estimated $\beta_{a,br}$ and $\beta_{a,bo}$ using Equation (4a/b) for all datasets used in Rogell et al. (2019), and compared them to simple and multiple regressions (Figure 4). There is little correlation between simple regressions and the direct‐effect estimates that fully account for the possibility of correlated evolution of both traits (Figure 4A,B). Partial effects from multiple regression analyses regressing each trait, in turn, on the selective agent and the other trait tends to agree in sign with the direct‐effect estimates that fully account for correlated evolution of both traits (Figure 4C,D). The mathematics of partial regression and the estimators of Equation (4a/b) do not require the two quantities to take the same signs, so the finding that their signs generally coincide may indicate that previous results from multiple regression may generally be qualitatively robust despite the risk of bias as illustrated in Figure 2B.

Some estimates of direct effects using Equation (4a/b) are very large (note the substantial vertical spread in Fig. 4). We caution against any conclusion that effects of putative selective agents on brain size and body size are as large as these. These estimates will come with very high uncertainty (Fig. 3). Summary statistics used in Rogell et al. (2019) consist of sample sizes and covariance matrices among selective agents and brain and body size. Therefore, while methods like case bootstrapping for generating standard errors may be useful to generate standard errors in applications of Equation (4a/b), such an approach cannot be applied in the present analysis. We have, however, calculated standard errors using an approach involving generating random samples from the estimated covariance matrices, which should give some idea of uncertainties in our estimates of direct effects that account for reciprocal correlated evolution. Investigation of these uncertainties is presented in Supporting information.

Earlier, we discussed how the partial regressions of selective agents on brain size and body size do not necessarily estimate direct effects of selective agents on the traits. However, the quantities generated by the “reversed” multiple regression analysis very nearly coincide with the direct‐effect estimates that allow for reciprocal correlated evolution. We show this in the Supporting information (page 19). This near‐alignment is perhaps unsurprising, because both analyses, in essence, tackle the problem of separating agent‐trait associations where the correlation between the traits is the main confounding factor. We note, however, that the coincidence of coefficients from the “reversed” multiple regression analysis and the estimators in Equation (4a/b) only holds when traits are standardized to have a common variance. This particular form of standardization is a feature of the (Rogell et al. 2019) summary statistics, and therefore, of our reanalyses, but is generally undesirable for comparative analyses, as it obscures the useful property of simple regression to reflect proportional changes when body size and focal traits, such as brain size, are expressed on the logarithmic scale.

### AVENUES FOR FURTHER DEVELOPMENT

We present the estimators of effects of selective agents on focal and potentially confounding traits, under a basic model of correlated responses to selection, as a step toward a more complete method to identify unbiased estimates of the diversification of allometric traits. By no means should this be interpreted as a fail‐safe solution to the problems we identify, which will require further study and methodological development. The estimators in Equation (4a/b) draw upon the simplest general model of correlated responses to selection, and may be seen as the most straightforward alternative for comparative analyses that would otherwise use multiple regression, but wish to avoid the biases that might arise under the simple model of reciprocal correlated evolution. Further developments could potentially incorporate additional phenomena, for example, that different taxa within an analysis might have different static allometries (as developed above, the analysis draws on an assumption of a single static allometry). Such an extension might, for example, yield a way of modeling the evolution of nonmonotonic allometric relationships. It is not clear if the MoM estimators accounting for reciprocal evolution could easily be extended to accommodate more complex situations such as variable static allometries. However, we have also implemented the basic model in a Bayesian framework, obtaining comparable solutions to the MoM estimators on simulated data (see Supporting information), and we suggest that this might serve as an approach that is conducive to further development. It is worth noting that Ornstein–Uhlenbeck models of trait evolution of allometric traits implemented in the R package SLOUCH (see Hansen et al. 2008), will inherently account for this problem as well.

Our analysis demonstrates that the causal models accommodated by simple and multiple regression, while not biased by collinearity, can nonetheless be biased for a different reason. Further extension of the basic reasoning will be necessary to handle more general cases, for example, multiple selective agents, and the fact that many of the selective agents of interest to biologists might themselves evolve as correlated responses to changes in body size and focal traits (see also, for example, discussion on variables such as longevity, which may lead to collider bias given the possible bidirectional links with brain size; McElreath 2020).

### PART 2: KEY POINTS

We suggest that the basic elements of a comparative analysis seeking to estimate direct effects of selective agents on focal traits, such as brain size, taking an allometric view into account, will likely want to consider two paths of possible indirect association. First, brain size may be indirectly associated with a selective agent, if it has evolved as a correlated response to adaptive evolution of body size in response to the agent. Second, body size may also evolve in an indirect manner, as a consequence of adaptive diversification of brain size. If these two indirect contributions to associations among relevant variables are plausible, then multiple regression does not necessarily recover direct effects of selective agents on focal traits. However, this divergence between multiple regression and the desired biological quantities does not arise because of collinearity among any variables in the analysis. Rather, it arises because multiple regression does not accommodate the causal mechanisms by which associations may arise. Our suggestion of estimators that do account for the main elements of more general model of potential reasons for associations of selective agents, body size, and focal traits should be seen as an illustration of a potential way forward if researchers want to account for the more general model of indirect associations.

## Conclusion

Regression models, including those estimating drivers of the diversification of allometric traits, are not immune to bias and deserve continued scrutiny. However, opposite‐signed estimates of simple and partial regression coefficients are not a problem in and of themselves. Rather, comparative studies estimating the evolution of allometric traits are susceptible to bias when regression analyses conflict with plausible causal structures. We first show that the construction of regression models requires that decisions about the treatment of different variables as predictors and responses must be biologically motivated. Then, we show that covariance between predictor variables, in and of itself, does not cause bias in multiple regression. Finally, we show that correlated responses to selection make models estimating the evolution of allometric traits particularly vulnerable to bias for separate reasons. It seems likely that bias arising from the use of regression models that are inconsistent with basic evolutionary processes like correlated responses to selection may be more common than is currently appreciated. We hope that the examples presented here, while not a complete review of the possible scenarios, highlight the importance of considering causal structures in comparative biology.

## AUTHOR CONTRIBUTIONS

SFW and MBM conceived and designed the study. MBM led the derivation of the proposed estimation equations. Both authors contributed to drafting and editing the manuscript.

## Supporting information

Supplementary information

## Data Availability

The summary statistics used in Rogell et al. (2019) are openly available on the *Evolution Letters* website (https://onlinelibrary.wiley.com/doi/full/10.1002/evl3.151). All simulated data used in the present article's analyses are generated using scripts presented in our Supporting Information.

## Footnotes

1 Partial regression coefficients match the estimates generated from a simple regression with relative brain size, but the multiple regression analysis should be preferred for more efficiently using the data and correctly representing uncertainty (Freckleton 2002; McElreath 2020).
