# Supplementary material for: Causation, not collinearity: Identifying sources of bias when modelling the evolution of brain size and other allometric traits
Source: Evol Lett. 2021 Nov 9;6(3):234–44. doi: 10.1002/evl3.258 (PMC9233177; doi:10.1002/evl3.258)
Supplement: Supplementary file 1 — Supplementary information [file EVL3-6-234-s001.pdf]

Supplementary analyses of brain body size associations

Sam F. Walmsley and Michael Morrissey

19 October, 2021

Numerical example 1: association without direct effect

The first example in the main text to illustrate how simple and partial regression coefficients can be different from one another, but nonetheless both unbiased involved a qualitative description consistent with this quantitative causal scheme:

```
library(mvtnorm)
n<-500
agent<-rnorm(n)
body<-rnorm(n,0.5*agent,1)
brain<-rnorm(n,0.5*body,1)
```

i.e., there is no direct effect of the focal agent on brain size. If it were not for the fact that brain size changes with body size (whether body size changes because of the agent or not), there would be no association of the agent with brain size. But the agent does affect body size, and body size does in turn affect brain size. The simple regression documents this overall association

```
summary(lm(brain~agent))$coefficients
```

```
##               Estimate Std. Error   t value     Pr(>|t|)
## (Intercept) 0.01477657 0.04925301 0.3000135 7.642920e-01
## agent       0.22132807 0.04490886 4.9283826 1.130803e-06
```

The multiple regression of brain size on the selective agent in body size reveals the other critical part of the story, namely, that there is no effect of the agent on brain size, over and above any knock-on effect of the agents direct effect on body size:

```
summary(lm(brain~agent+body))$coefficients
```

```
##                Estimate Std. Error    t value     Pr(>|t|)
## (Intercept)  0.05143957 0.04434860  1.1598914 2.466499e-01
## agent       -0.01097292 0.04552875 -0.2410108 8.096462e-01
## body         0.46847800 0.04263608 10.9878305 2.723858e-25
```

Numerical example 2: sign reversals

The second verbal illustration in the main text describes a situation where the direct and indirect contributions to the association of the selective agent and brain size have opposite signs, such that it is possible for the direct effect and overall associations to have different signs. A qualitative scheme that generates the different signs is this:

```
n<-500
agent<-rnorm(n)
body<-rnorm(n,0.5*agent,sqrt(0.75))
brain<-rnorm(n,-0.1*agent+0.5*body,1)
```

The strong effects of the agent on body size, and of body size on brain size drive the overall association, which is positive:

```
summary(lm(brain~agent))$coefficients
```

```
##                Estimate Std. Error    t value  Pr(>|t|)
## (Intercept) -0.01043335 0.04717413 -0.2211668 0.8250532
## agent        0.05985810 0.04626483  1.2938145 0.1963292
```

However, over and above any indirect effect that the selective agent has via body size, we did simulate a smaller, negative, direct effect (of -0.1 units of brain size per unit of the selective agent). The multiple regression picks up this direct effect:

```
summary(lm(brain~agent+body))$coefficients
```

```
##                Estimate Std. Error    t value     Pr(>|t|)
## (Intercept)  0.01998629 0.04301852  0.4645972 6.424234e-01
## agent       -0.14155207 0.04646252 -3.0465864 2.437600e-03
## body         0.50334304 0.04918359 10.2339621 1.957494e-22
```

Unbiasedness of both simple and partial regression coefficients

The main text describes how various quantitative observations, particularly that signs of simple and partial regression coefficients can differ, are not evidence that either are valid. This is not the same as demonstrating that they are unbiased. This section simulates multiple regression with collinear predictor variables, and shows that multiple regression recovers true effects, on average (of course, it doesn't ever get exactly the right answer, due to finite sample size). In the section on reversibility of regression analyses, we focused on the importance of what variable is treated as the response variable, particularly in multiple regression analysis. We provide code for using arbitrary sample sizes, correlations among predictors, and true effects, but only run it for a single case. Readers can change any key parameters to verify for themselves that the analysis is unbiased by correlations among predictor variables. More comprehensive ranges of simpualtion paramters are presented in Morrissey and Ruxton (2018).

```
# simulation paramters
n<-100    # sample size for each simulation
n.sim<-1000 # number of replicate simulations
rho<-0.8 # correlation between two predictor variables
beta_1<-0.25 # partial effet of first predictor variable
beta_2<- -0.5 # partial effet of second predictor variable
```

```
# a place to save the estimates from replicate simulations
res<-array(dim=c(n.sim,2))

# loop over replicate simulations
for(i in 1:n.sim){
```

```
  # simulate data
  x<-rmvnorm(n,c(0,0),matrix(c(1,rho,rho,1),2,2))
  x1<-x[,1]
  x2<-x[,2]
  y<-beta_1*x1+beta_2*x2+rnorm(n)

  # conduct analysis and save results for the two
  # partial regression coefficients
  res[i,]<-coef(lm(y~x1+x2))[2:3]
}
```

If multiple regression was unbiased, regardless of whatever correlation between predictor variables, then bias, or the difference between the expectation (long-run average value) of the estimator and the true parameter value, should be zero:

```
# average estimate for first regression coefficient
mean(res[,1])
```

```
## [1] 0.2510753
```

```
# bias of first regression coefficient
mean(res[,1])-beta_1
```

```
## [1] 0.001075268
```

```
# average estimate for second regression coefficient
mean(res[,2])
```

```
## [1] -0.5107007
```

```
# bias of second regression coefficient
mean(res[,2])-beta_2
```

```
## [1] -0.01070071
```

Numerical example 3: model misspecification error if a consequence is treated as a cause

Say some variable x affects another variable y, and y, in turn, affects z. One might think that it would be safest to inlcude z if it seems it might be correlated with x and y, if one wanted to know the effect of x on y. Unfortunately, this is not necessarily safe. If variation in z is a consequence of variation in y, then a multiple regression of y on x and z will be inconsistent with the data-generating process in such a way that leads to an error due to model mis-specification:

```
n<-1000
x<-rnorm(n)
y<-rnorm(n,0.5*x,sqrt(0.75))
z<-rnorm(n,0.5*y,sqrt(0.75))
```

the mis-specified regression model is

```
summary(lm(y~x+z))$coefficients
```

```
##               Estimate Std. Error   t value     Pr(>|t|)
## (Intercept) 0.02710943 0.02462533  1.100876 2.712163e-01
## x           0.38334019 0.02488790 15.402673 3.509102e-48
## z           0.42076978 0.02485510 16.928914 1.061439e-56
```

The multiple regression that correctly recovers the direct effect of x on y is the model that includes all variables that contribute to the overall association of x and y. In this simple case, this is the regression of y on x only

```
summary(lm(y~x))$coefficients
```

```
##               Estimate Std. Error    t value     Pr(>|t|)
## (Intercept) 0.01552083 0.02791655  0.5559725 5.783543e-01
## x           0.48537472 0.02738493 17.7241538 2.537616e-61
```

In this simple causal model, the correctly specified multiple regression model coincides with the simple regression.

Numerical example 4: correlated responses to selection mean that traits are both causes and consequences of each other

Suppose that, if it were not for some focal selective agent, brain size and body size had an allometric relationship with a slope of 0.8, and with a regresion of body size on brain size of 0.4:

```
n<-10000
brain_body<-rmvnorm(n,c(1,1),matrix(c(4,1.6,1.6,2),2,2))
brain0<-brain_body[,1]; body0<-brain_body[,2];
```

by `brain0` and `body0` we mean the values that brain size and body size would be, before accounting for the evolutionary effects of a focal selective agent. We can verify that the simulation recovers the regressions described above:

```
summary(lm(brain0~body0))$coefficients
```

```
##              Estimate Std. Error   t value    Pr(>|t|)
## (Intercept) 0.1777387 0.02044566  8.693223 4.07614e-18
## body0       0.7898697 0.01168398 67.602772 0.00000e+00
```

```
summary(lm(body0~brain0))$coefficients
```

```
##              Estimate  Std. Error  t value Pr(>|t|)
## (Intercept) 0.6316317 0.013110187 48.17870        0
## brain0      0.3971637 0.005874962 67.60277        0
```

Now, suppose that some selective agent

```
agent<-rnorm(n)
```

has a direct an effect on brain size of -0.2 (units of brain size per unit of selective agent). Under the standing allometric relationship, we would expect a correlated response in brain size, according to the allometric relationship.

```
brain_direct  <- -0.2*agent
body_indirect <- -0.2*agent*0.4
```

Suppose that the selective agent has an effect on body size of +0.4. We would expect body size to also change accordingly, according to the regression of body size on brain size. Putting both together

```
body_direct    <- 0.4*agent
brain_indirect <- 0.4*agent*0.8
```

Putting all the effects together and adding them on top of the background allometry gives us:

```
body <- body0 + body_direct + body_indirect
brain <-brain0 + brain_direct + brain_indirect
```

In this scenario, the multiple regression analysis does not correctly recover the direct effects of the agent on brain size (-0.2) and of the agent on body size (+0.4):

```
summary(lm(brain~agent+body))$coefficients[2,]
```

```
##      Estimate    Std. Error       t value      Pr(>|t|)
## -1.235858e-01  1.694822e-02 -7.291961e+00  3.286366e-13
```

```
summary(lm(body~agent+brain))$coefficients[2,]
```

```
##      Estimate    Std. Error       t value      Pr(>|t|)
##  2.629416e-01  1.175921e-02  2.236048e+01 4.152744e-108
```

Here is a function that applies equations 4a and 4b, when provided with a covariance matrix of the agent, the size variablem, and the focal trait, in that order:

```
MoM_estimators<-function(Sigma_obs){
  VARa<-Sigma_obs[1,1]
  VARbo<-Sigma_obs[2,2]
  VARbr<-Sigma_obs[3,3]
  COVabo<-Sigma_obs[1,2]
  COVabr<-Sigma_obs[1,3]
  COVbrbo<-Sigma_obs[2,3]

  beta_brain<- -((COVabo*COVbrbo-COVabr*VARbo)*(COVabr^2-VARa*VARbr))/
     (VARa*(-2*COVabo*COVabr*COVbrbo+COVbrbo^2*VARa+COVabr^2*VARbo+
              COVabo^2*VARbr-VARa*VARbo*VARbr))

  beta_body<- -((-COVabr*COVbrbo+COVabo*VARbr)*(COVabo^2-VARa*VARbo))/
     (VARa*(2*COVabo*COVabr*COVbrbo-COVbrbo^2*VARa-COVabr^2*VARbo-
```

```
              COVabo^2*VARbr+VARa*VARbo*VARbr))

  return(c(agent_brain_effect=beta_brain,agent_body_effect=beta_body))
}
```

These estimators recover the correct values (-0.2 and +0.4):

```
MoM_estimators(cov(cbind(agent,body,brain)))
```

```
## agent_brain_effect  agent_body_effect
##         -0.1800827          0.3831446
```

Verification of unbiasedness, and investigation of precision, of estimation accounting for correlated evolution of both phenotypes

This section provides the code to generate figure 3, plus an additional figure showing a further range of possible outcomes.

This function does one replicate of our simulation scenario. It generates traits based on (a) the underlying allometric relationship, and then (b) superimposes the direct and indirect diversification resulting from effects of a selective agent on both body size and brain size.

```
sim_estimators<-function(beta_abr=0,beta_abo=0,sigma=diag(2),
                         var_agent=1,n=20,n.sim=1000){
  res<-array(dim=c(n.sim,3,2))
  for(i in 1:n.sim){
    # start constructing a data frame; first make up records
    # of brain and body size before the selective agent becomes
    # relevant
    d<-rmvnorm(n,c(3,2), sigma)
    d<-data.frame(body=d[,1],brain=d[,2])
    # here is a selective agent
    d$agent<-rnorm(n,0,sqrt(var_agent))
    # selective agent acts on body size; direct and indirect components
    d$new.body<- d$body + beta_abo*d$agent + sigma[1,2]/sigma[2,2] * beta_abr*d$agent
    # same for evolution of brain size
    d$new.brain <- d$brain + beta_abr*d$agent  + sigma[1,2]/sigma[1,1] * beta_abo*d$agent
    # store results
    res[i,1,1]<-coef(lm(new.brain~agent,data=d))[2]
    res[i,1,2]<-coef(lm(new.body~agent,data=d))[2]
    res[i,2,1]<-coef(lm(new.brain~agent+new.body,data=d))[2]
    res[i,2,2]<-coef(lm(new.body~agent+new.brain,data=d))[2]
    res[i,3,]<-MoM_estimators(cov(d[,c("agent","new.body","new.brain")]))
  }
  return(res)
}
```

This runs the above core simulation function for the range of variables described in the main text.

```
n_vals<-c(20,50,100,200)
corr_vals<-c(0.5,0.8,0.9)
beta_abr_vals<-seq(-0.5,0.5,length.out=20)
```

```
# dimensions for results array:
#   - sample size
#   - background allometric correlation
#   - agent-brain true values
#   - simple, partial, and full model
#   - simulation expectation and SE
sim_res<-array(dim=c(4,3,20,3,2))

# doing the simulations: this takes about fifteen minuters; if a saved results file
# exists in the working directory, the results will be used and the simulation
# skipped (apologies to RMarkdown purists for the poor-man's results caching)
if("agent_brain_diversification_sims.RData" %in% system("ls",intern=TRUE)){
  load("./agent_brain_diversification_sims.RData")
}else{
  for(i in 1:4){
    for(j in 1:3){
      for(k in 1:20){
        r<-sim_estimators(beta_abr=beta_abr_vals[k],
                beta_abo=0.2,sigma=matrix(c(1,corr_vals[j],
                                             corr_vals[j],1),2,2),
                n=n_vals[i],n.sim=100)
        sim_res[i,j,k,,1]<-apply(r[,,1],2,mean)
        sim_res[i,j,k,,2]<-apply(r[,,1],2,sd)
      }
    }
  } # end simulations
} # end if
```

Save the results, so they don't necessarily have to be re-run every time time the supplement is compiled.

```
if(TRUE){
  save(sim_res,file="./agent_brain_diversification_sims.RData")
}
```

A helper function for making the subplots in main text figure 3, and also for the supplementary figure below.

```
mk_plt<-function(nval,corval,offset=0.01,sim_res,...){
  plot(beta_abr_vals-offset,sim_res[nval,corval,,1,1],pch=4,...,
      col="red",ylim=c(-1,1),xaxt='n',yaxt='n')
  points(beta_abr_vals+offset,sim_res[nval,corval,,2,1],pch=5,col="blue")
  points(beta_abr_vals,sim_res[nval,corval,,3,1])
  for(i in 1:20){
    lines(rep(beta_abr_vals[i]-offset,2),
          sim_res[nval,corval,i,1,1]+c(-1,1)*
            sim_res[nval,corval,i,1,2],col="red")
    lines(rep(beta_abr_vals[i]+offset,2),
          sim_res[nval,corval,i,2,1]+c(-1,1)*
            sim_res[nval,corval,i,2,2],col="blue")
    lines(rep(beta_abr_vals[i],2),
          sim_res[nval,corval,i,3,1]+c(-1,1)*
            sim_res[nval,corval,i,3,2])
  }
```

```
  abline(0,1,col="green")
}
```

118 Code to generate figure 3.

```
par(mfrow=c(4,3),mar=c(0.5,0.5,0.5,0.5),oma=c(4,4,1.5,1),las=1)
for(i in 1:4){
  for(j in 1:3){
    mk_plt(i,j,sim_res=sim_res)
    if(i==1) mtext(side=3,line=0.5,bquote(rho~" ="~.(corr_vals[j])))
    if(j==3) mtext(side=4,line=0.5,bquote("n ="~.(n_vals[i])),las=0)
    if(i<4){
      axis(side=1,seq(-0.5,0.5,length.out=5),rep("",5))
    }else{
      axis(side=1,seq(-0.5,0.5,length.out=5))
    }
    if(j==1){
      axis(side=2,seq(-1,1,length.out=5))
    }else{
      axis(side=2,seq(-1,1,length.out=5),rep("",5))
    }
    if(i==4&j==1) legend("topleft",bty='n',
                col=c("red","blue","black","green"),
                pch=c(4,5,21,NA),lty=c(NA,NA,NA,"solid"),
                legend=c("simple regression","partial regression",
                         "MoM estimation","1:1"))
  }
}
mtext(side=1,outer=TRUE,"true effect of agent on brain size",line=2.5)
mtext(side=2,outer=TRUE,"parameter simulation mean and SE",las=0,line=2.5)
```

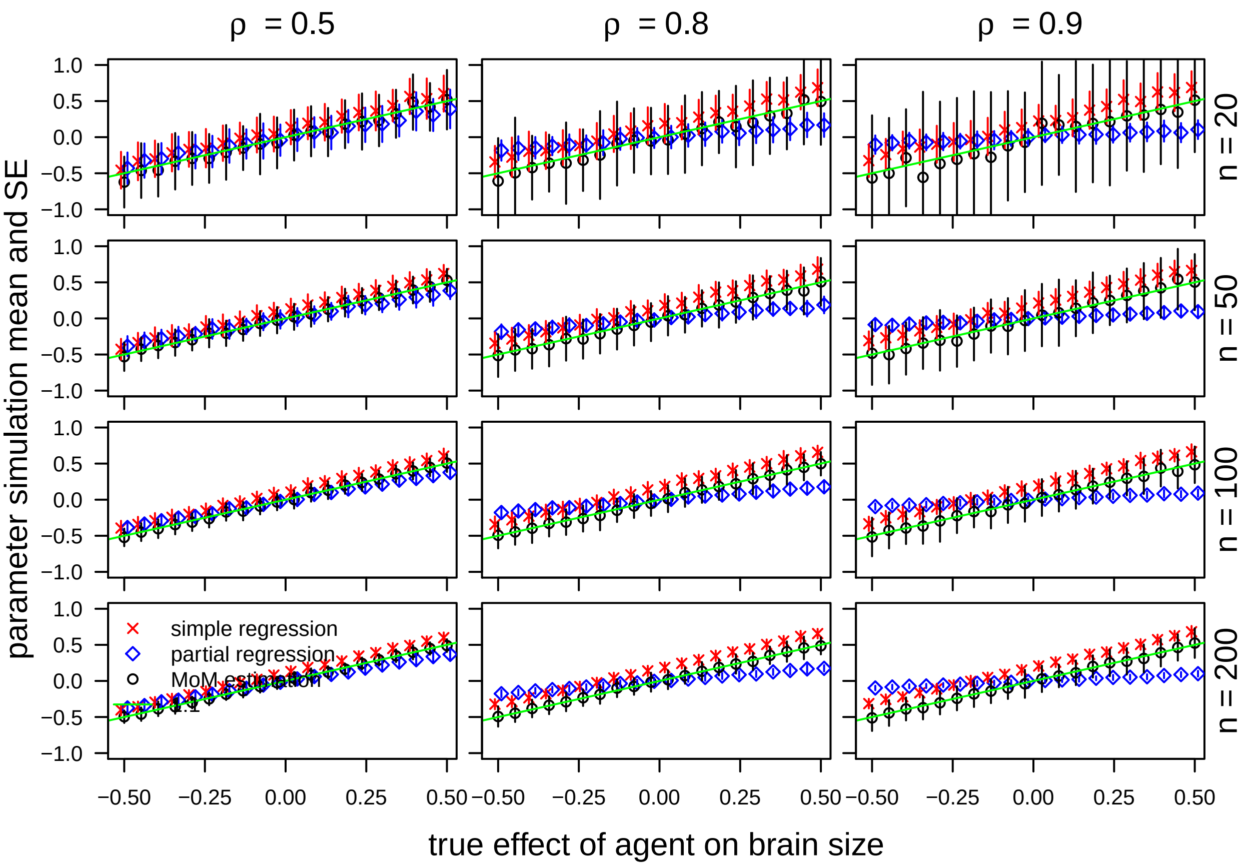

The intention of figure 3 is not to provide a comprehensive simulation study, but rather to demonstrate the potenital estimability of direct effects under the more general model of correlated evolutionary responses. Concequently, figure 3 does not necessarily illustrate all possible contrasts that can occur between the different sorts of coefficients to characterise assocations and effects. This simulation is much the same, with the only difference being a negative direct effect of the selective agent on body size.

```
n_vals<-c(20,50,100,200)
corr_vals<-c(0.5,0.8,0.9)
beta_abr_vals<-seq(-0.5,0.5,length.out=20)

# dimensions for results array:
#   - sample size
#   - background allometric correlation
#   - agent-brain true values
#   - simple, partial, and full model
#   - simulation expectation and SE
sim_res_alt<-array(dim=c(4,3,20,3,2))

# doing the simulations: this takes about fifteen minuters; if a saved results file
# exists in the working directory, the results will be used and the simulation
# skipped (apologies to RMarkdown purists for the poor-man's results caching)
if("agent_brain_diversification_sims_alt.RData" %in% system("ls",intern=TRUE)){
  load("./agent_brain_diversification_sims_alt.RData")
}else{
  for(i in 1:4){
    for(j in 1:3){
```

```
      for(k in 1:20){
        r<-sim_estimators(beta_abr=beta_abr_vals[k],
                beta_abo=-0.2,sigma=matrix(c(1,corr_vals[j],
                                            corr_vals[j],1),2,2),
                n=n_vals[i],n.sim=100)
        sim_res_alt[i,j,k,,1]<-apply(r[,,1],2,mean)
        sim_res_alt[i,j,k,,2]<-apply(r[,,1],2,sd)
      }
    }
  } # end simulations
} # end if
```

Again, save for convenience.

```
if(TRUE){
  save(sim_res_alt,file="./agent_brain_diversification_sims_alt.RData")
}
```

Here is an analog of figure 3, but for the negative direct effect of the agent on body size. Some of the possible further outcomes are illustrated, in particular, the overall associations are now generally smaller (more negative) than the direct effects.

```
par(mfrow=c(4,3),mar=c(0.5,0.5,0.5,0.5),oma=c(4,4,1.5,1),las=1)
for(i in 1:4){
  for(j in 1:3){
    mk_plt(i,j,sim_res=sim_res_alt)
    if(i==1) mtext(side=3,line=0.5,bquote(rho~" ="~.(corr_vals[j])))
    if(j==3) mtext(side=4,line=0.5,bquote("n ="~.(n_vals[i])),las=0)
    if(i<4){
      axis(side=1,seq(-0.5,0.5,length.out=5),rep("",5))
    }else{
      axis(side=1,seq(-0.5,0.5,length.out=5))
    }
    if(j==1){
      axis(side=2,seq(-1,1,length.out=5))
    }else{
      axis(side=2,seq(-1,1,length.out=5),rep("",5))
    }
    if(i==4&j==1) legend("topleft",bty='n',
               col=c("red","blue","black","green"),
               pch=c(4,5,21,NA),lty=c(NA,NA,NA,"solid"),
               legend=c("simple regression","partial regression",
                        "MoM estimation","1:1"))
  }
}
mtext(side=1,outer=TRUE,"true effect of agent on brain size",line=2.5)
mtext(side=2,outer=TRUE,"parameter simulation mean and SE",las=0,line=2.5)
```

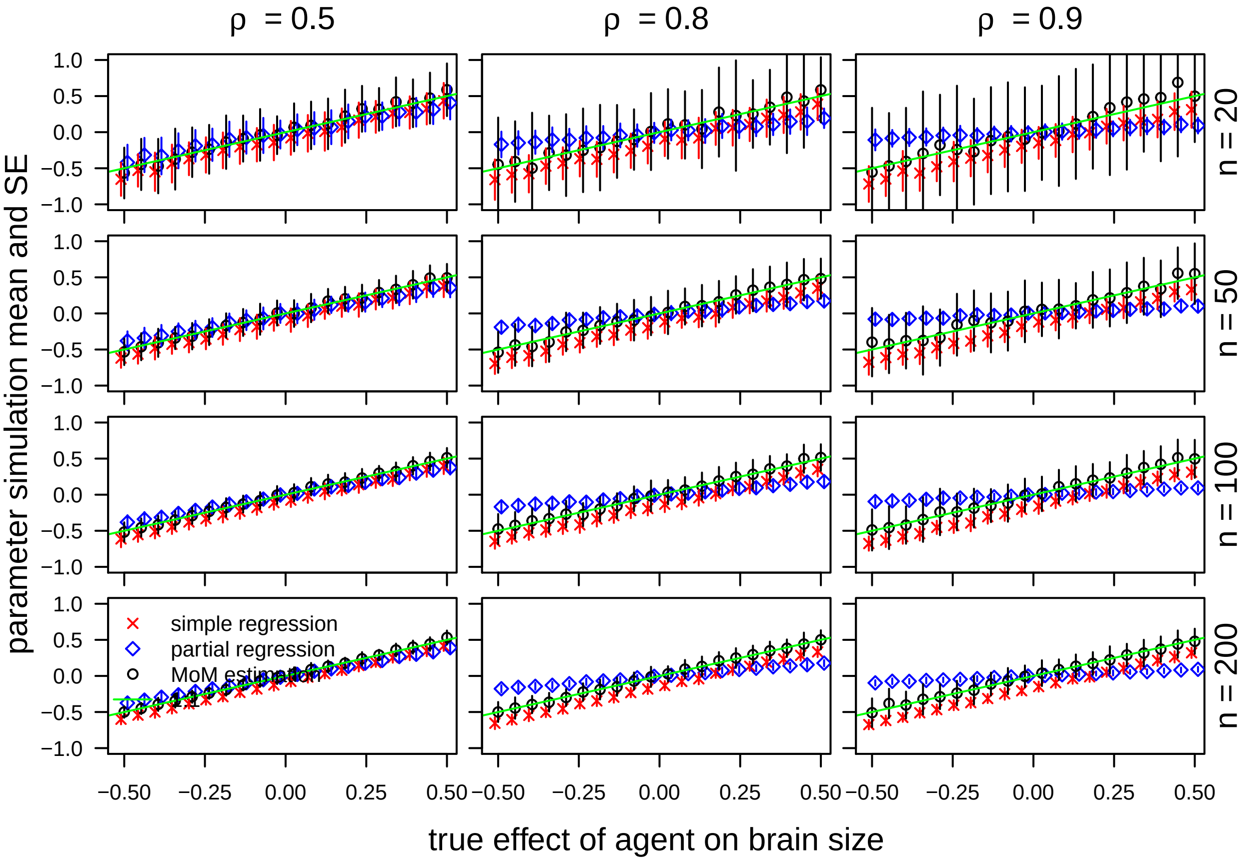

“Reversability” of multiple regression analyses

In the section “The treatment of predictor and response variables in regression models’ ’ we explain that the coefficients estimated by the multiple regression of an hypothesised selective agen on brain and body size, i.e., of the form

$$agent_i = \alpha + \beta_{brain\ eq.1} \cdot brain_i + \beta_{body\ eq.1} \cdot body_i + e_i \tag{1}$$

do not coincide with the partial regressions obtained from separate multiple regressions of the form

$$brain_i = \alpha + \beta_{brain\ eq.2a} agent_i \cdot \beta_{body\ eq.2a} \cdot body_i + e_i \tag{2a}$$

$$body_i = \alpha + \beta_{body\ eq.2b} agent_i \cdot \beta_{brain\ eq.2b} \cdot brain_i + e_i \tag{2b}$$

Specifically $\beta_{brain\ eq.1}$ does not estimate the effect of the selective agent on brain size, controlling for body size (as does $\beta_{brain\ eq.2a}$), and $\beta_{body\ eq.1}$ does not estimate the effect of the selective agen on body size, controlling for brain size (as does $\beta_{body\ eq.2b}$). In this section we provide a toy model to illustrate the differences.

Numerical example 5

Suppose a simple scenario where effects of body size may affect the evolution of brain size, but brain size does not have any effect on body size. Furthermore suppose that body size and the selective agent are correlated with $\rho = 0.9$:

```
n<-5000
rho<-0.9
Sigma<-matrix(c(1,rho,rho,1),2,2)
agent_body<-rmvnorm(n,c(0,0),Sigma)
agent<-agent_body[,1]
body<-agent_body[,2]
```

Now suppose that the selective agent acts such that, in itself, an increase of the value of the agent of one unit generates, on average and independently of any associative effects acting via body size, an increase of brain size of 0.2 units. Suppose, furthermore, that an increase in body size, generates, on averageand independently of any associative effects acting via the selective agent, an increase of brain size of 0.4 units.

```
beta_agent_true<-0.2
beta_body_true<-0.4
sigma<-sqrt(1-c(beta_agent_true,beta_body_true)%*%Sigma%*%
                 c(beta_agent_true,beta_body_true))
brain<-beta_agent_true*agent+beta_body_true*body+rnorm(n,0,sigma)
```

The simulation of the residual SD in brain size is made such that the expercted total variance of brain size will be one. This is not, in general, critical to understanding how multiple regression works, but it closely follows the standardisations made by Rogell et al (2019) in the course of their various simple and multiple regression analyses.

We now have a system in which there are associations among all three variables

```
cov(cbind(agent,brain,body))
```

```
##           agent     brain      body
## agent 0.9654711 0.5291656 0.8725536
## brain 0.5291656 0.9912418 0.5564412
## body  0.8725536 0.5564412 0.9744987
```

Under variance standardisation (note that all variables have a variance of one), simple regressions (i.e., a single predictor variable) recover these correlations, regardless of which variable is the predictor variable:

```
lm(agent~brain)
```

```
##
## Call:
## lm(formula = agent ~ brain)
##
## Coefficients:
## (Intercept)        brain
##   -0.009454     0.533841
```

```
lm(brain~agent)
```

```
##
## Call:
## lm(formula = brain ~ agent)
##
## Coefficients:
## (Intercept)        agent
##   -0.008344     0.548091
```

However, the multiple regressions are not “reversible” in this way:

```
# Rogell et al model for recovering the partial effects:
coef(lm(agent~brain+body))[2:3]
```

```
##      brain       body
## 0.04593123 0.86916030
```

these coefficients were taken to be equivalent to these coefficients

```
coef(lm(brain~agent+body))[2]
```

```
##     agent
## 0.1679459
```

```
coef(lm(body~agent+brain))[2]
```

```
##     agent
## 0.8426335
```

which they are not. The former are the multiple regressions that are relavent to understanding, for example, the effect of a selective agent on brain size, controlling for any associations arising through effects of body size.

How does calculating the correct partial regressions change the results?

Here are the data from Rogel et al

```
d<-read.table("./Rogell_et_al_data.csv",header=TRUE,sep=',')
```

First, let’s have a look at the simple regressions, which should exactly reflect the corresponding correlations, as these are identical when all variable have unit variance

```
add_guide_lines<-function(){
  lines(c(-100,100),c(0,0),col="blue")
  lines(c(0,0),c(-100,100),col="blue")
  lines(c(-100,100),c(-100,100),col="red")
}


par(mfrow=c(1,2))
plot(d$v.brain.corr,d$univariate.brain)
add_guide_lines()
plot(d$v.body.corr,d$univariate.body)
add_guide_lines()
```

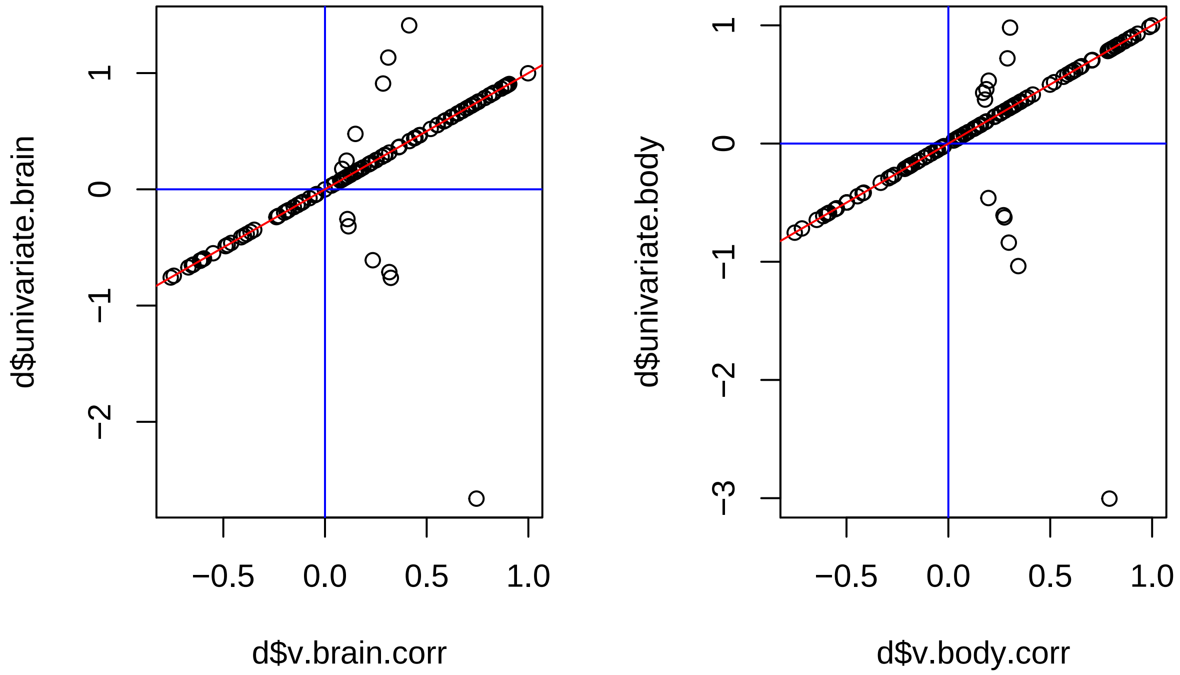

Why a few points don't fall exactly on the 1:1 line is not clear, but there must be errors associated with these entries. We will remove them:

```
d<-subset(d,d$v.brain.corr == d$univariate.brain)
```

For the remaining records, we will calculate the correct partial regression coefficients, as well as the effects that allow for correlated evolution:

```
for(i in 1:(dim(d)[1])){
  # partial effect of the agent on brain
  S<-matrix(c(1,d$v.body.corr[i],d$v.body.corr[i],1),2,2)
  C<-matrix(c(d$v.brain.corr[i],d$brain.body.corr[i]),2,1)
  beta<-(solve(S)%*%C)
  d$beta_abr[i]<-beta[1,1]
  s2<-as.numeric(1-t(beta)%*%S%*%beta)
  d$se_beta_abr[i]<-sqrt((s2*solve(d$sample.size[i]*S))[1,1])

  # partial effect of the agent on brain
  S<-matrix(c(1,d$v.brain.corr[i],d$v.brain.corr[i],1),2,2)
  C<-matrix(c(d$v.body.corr[i],d$brain.body.corr[i]),2,1)
  beta<-(solve(S)%*%C)
  d$beta_abo[i]<-beta[1,1]
  s2<-as.numeric(1-t(beta)%*%S%*%beta)
  d$se_beta_abo[i]<-sqrt((s2*solve(d$sample.size[i]*S))[1,1])

}
```

```
## Warning in sqrt((s2 * solve(d$sample.size[i] * S))[1, 1]): NaNs produced

## Warning in sqrt((s2 * solve(d$sample.size[i] * S))[1, 1]): NaNs produced

## Warning in sqrt((s2 * solve(d$sample.size[i] * S))[1, 1]): NaNs produced

## Warning in sqrt((s2 * solve(d$sample.size[i] * S))[1, 1]): NaNs produced
```

Plot to see how wrong the erroneous values were

```
par(mfrow=c(1,2))
plot(d$partial.brain,d$beta_abr)
add_guide_lines()
plot(d$partial.body,d$beta_abo)
add_guide_lines()
```

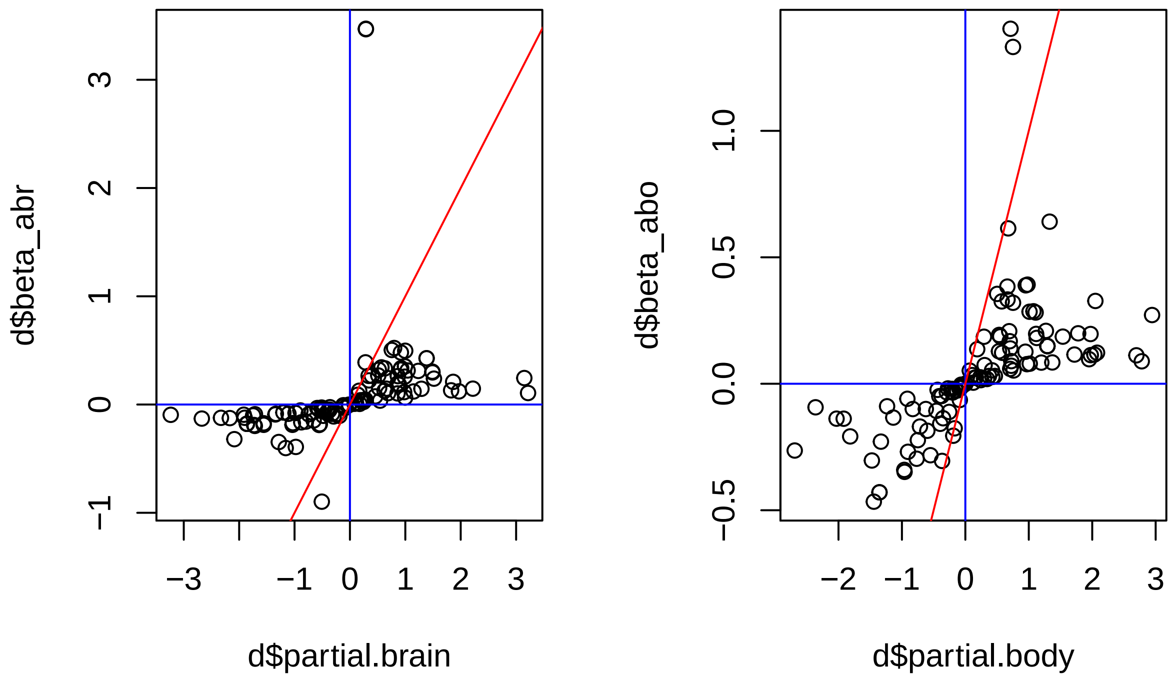

How does calculating the correct partial regression coefficients change our understanding of the correspondence (or lack thereof) between simple and partial regression coefficients:

```
par(mfrow=c(2,2))

dat<-d

plot(dat$partial.brain,dat$univariate.brain,
       xlab=expression(paste("erroneous ",beta[brain])),
       ylab="univariate brain regression")
```

```
add_guide_lines()
plot(dat$partial.body,dat$univariate.body,
       xlab=expression(paste("erroneous ",beta[body])),
       ylab="univariate body regression")
add_guide_lines()

plot(dat$beta_abr,dat$univariate.brain,
       xlab=expression(paste("correct ",beta[brain])),
       ylab="univariate brain regression")
add_guide_lines()
plot(dat$beta_abo,dat$univariate.body,
       xlab=expression(paste("correct ",beta[body])),
       ylab="univariate body regression")
add_guide_lines()
```

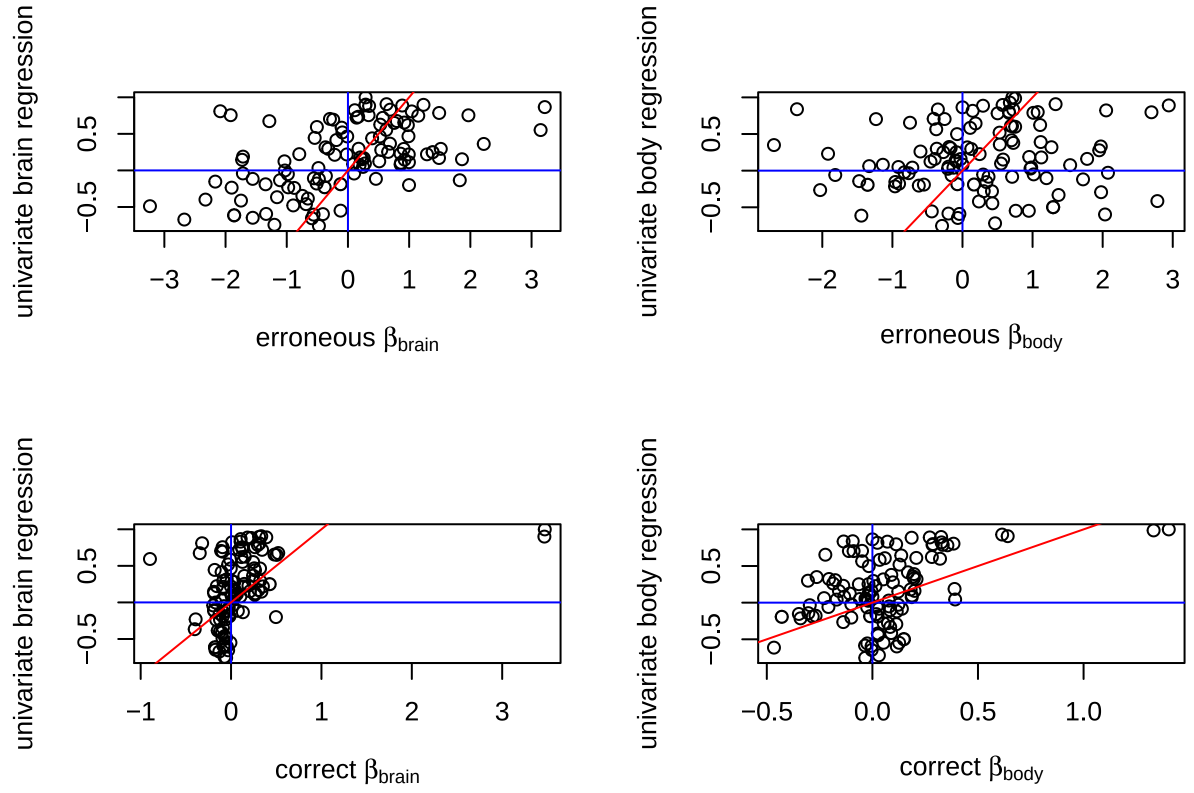

Make sure that this doesn't change vastly if we focus on just those studies with reasonable sample sizes:

```
par(mfrow=c(2,2))

cutoff<-50
dat<-subset(d,d$sample.size<=cutoff)

plot(dat$partial.brain,dat$univariate.brain,
     xlab=expression(paste("erroneous ",beta[brain])),
     ylab="univariate brain regression")
add_guide_lines()
```

```
plot(dat$partial.body,dat$univariate.body,
     xlab=expression(paste("erroneous ",beta[body])),
     ylab="univariate body regression")
add_guide_lines()

plot(dat$beta_abr,dat$univariate.brain,
     xlab=expression(paste("correct ",beta[brain])),
     ylab="univariate brain regression")
add_guide_lines()
plot(dat$beta_abo,dat$univariate.body,
     xlab=expression(paste("correct ",beta[body])),
     ylab="univariate body regression")
add_guide_lines()
```

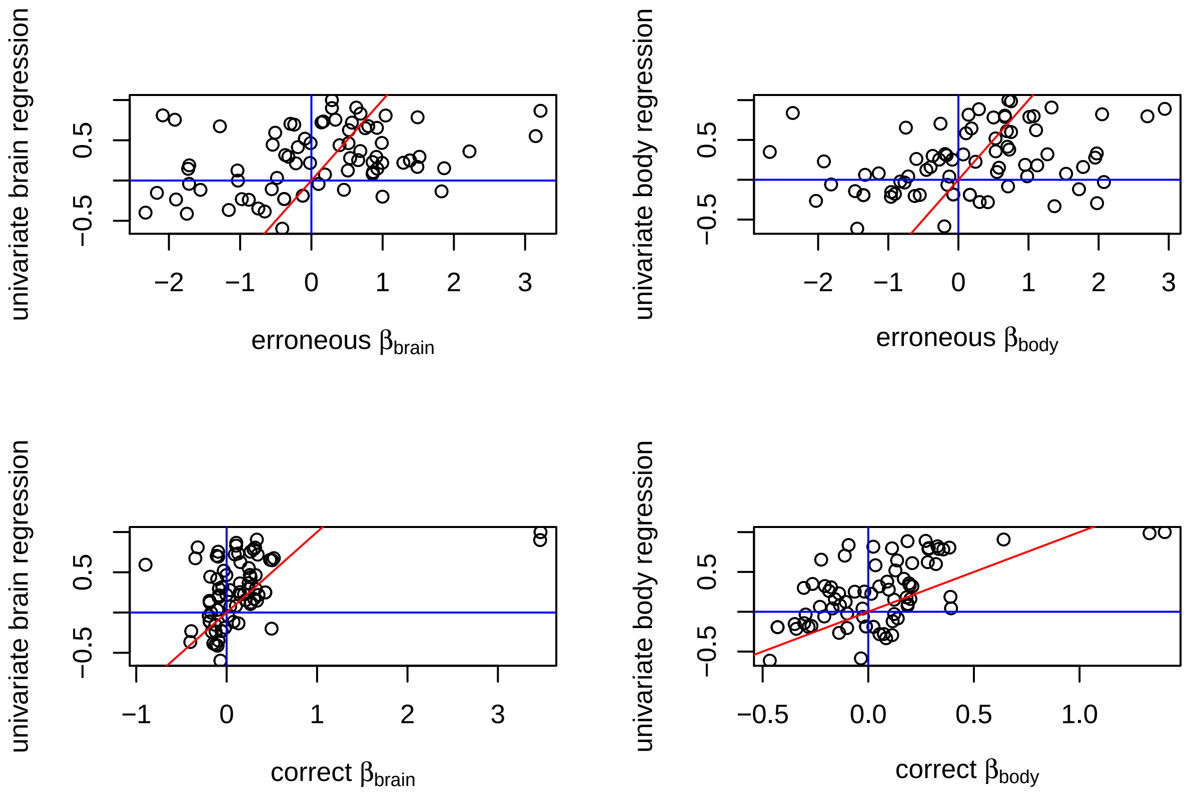

Effects of selective agents in the meta dataset, accounting for correlated evolution of both phenotypes

This section provides the code used to generate figure 4.

Loop through the dataset and generate the direct effect estimators that allow for a basic model of reciprocal correlated evolution:

```
d$effect_abr<-d$effect_abo<-NA
for(i in 1:(dim(d)[1])){
```

```
  c<-diag(3)
  c[1,2]<-c[2,1]<-d$v.body.corr[i]
  c[1,3]<-c[3,1]<-d$v.brain.corr[i]
  c[2,3]<-c[3,2]<-d$brain.body.corr[i]
  d[i,c("effect_abr","effect_abo")]<-MoM_estimators(c)
}
```

Plots of simple and multiple regressions against the direct effect estimators that allow for a basic model of reciprocal correlated evolution:

```
par(mfrow=c(2,2),mar=c(4,4,2,2),las=1)

add_guide_lines<-function(){
  lines(c(-100,100),c(0,0),col="blue")
  lines(c(0,0),c(-100,100),col="blue")
  lines(c(-100,100),c(-100,100),col="red")
}

max.plt.est<-4

with(d[which(abs(d$effect_abr)<max.plt.est),],
     plot(univariate.brain,effect_abr,xlab="simple regression",
          ylab="direct effect estimate",xlim=c(-1,1),ylim=c(-3,3)))
add_guide_lines()
mtext(side=3,outer=FALSE,line=-1.5,adj=0," (a)")
mtext(side=3,line=1,"agent/brain coefficients")
with(d[which(abs(d$effect_abo)<max.plt.est),],
     plot(univariate.body,effect_abo,xlab="simple regression",
          ylab="direct effect estimate",xlim=c(-1,1),ylim=c(-3,3)))
add_guide_lines()
mtext(side=3,outer=FALSE,line=-1.5,adj=0," (b)")
mtext(side=3,line=1,"agent/body coefficients")

with(d[which(abs(d$effect_abr)<max.plt.est),],
     plot(beta_abr,effect_abr,xlab="multiple regression",
          ylab="direct effect estimate",xlim=c(-1,1),ylim=c(-3,3)))
add_guide_lines()
mtext(side=3,outer=FALSE,line=-1.5,adj=0," (c)")
with(d[which(abs(d$effect_abo)<max.plt.est),],
     plot(beta_abo,effect_abo,xlab="multiple regression",
          ylab="direct effect estimate",xlim=c(-1,1),ylim=c(-3,3)))
add_guide_lines()
mtext(side=3,outer=FALSE,line=-1.5,adj=0," (d)")
```

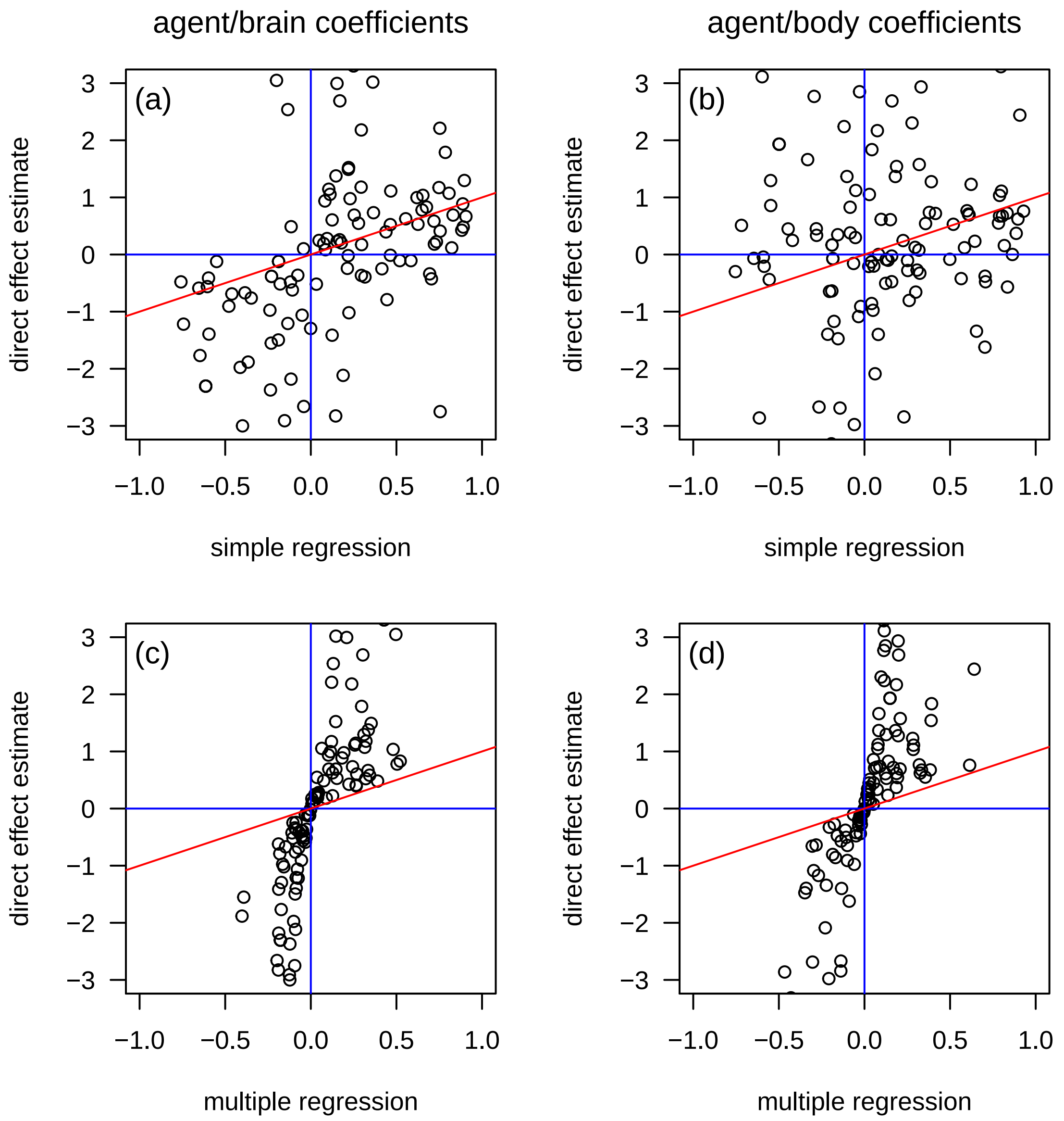

A preliminary scheme for generating standard errors for estimates that account for correlated evolution of both phenotypes

Given only sample size and estimated correlations among agents, brain size, and body size, analytical approximations for direct effect estimators that allow for a basic model of reciprocal correlated evolution will be extremely complex, as they will depend on sampling covaraínces among the corelation estimates. As a parallel to case bootstrapping, which would be applicable in an analysis of raw data, we generated pseudo-samples using each sample size and observed correlation matrix, and took the covariance of esimates based on these pseudo samples as an indication of the estimation error covariance matrix:

```
MC.se<-function(sigma,n,cor=FALSE,n.MC=1000){
  res<-array(dim=c(n.MC,2))
  for(i in 1:n.MC){
    r<-rmvnorm(n,c(0,0,0),sigma)
    if(cor==TRUE){
      ses<-apply(r,2,sd)
```

```
      r<-r/matrix(ses,n,3,byrow=TRUE)
    }
    res[i,]<-MoM_estimators(cov(r))
  }
  return(list(ses=apply(res,2,sd),rho=cor(res[,1],res[,2]),vcv=cov(res)))
}
```

Based on the simulations (figure 3), we expect extreme uncertainty for the smallest sample sizes and highest correlations between brain size and body size. We therefore proceed to illustrate the statistical uncertainty only for those cases where we can have some reasonable expectation of obtaining informative results. Specifically, we only considered studies with sample sizes above 40 and brain-body size correlations below 0.95:

```
d_sub<-subset(d,d$sample.size>40 & d$brain.body.corr<0.95)
dim(d_sub)
```

```
## [1] 15 18
```

We plot sampling error variance-covaraince matrices. We depict these as ellipses containing 95% if the the probability density of a bivariate normal distribution with the estimation error variances and covariance.

```
library(ellipse)
```

```
##
## Attaching package: 'ellipse'
```

```
## The following object is masked from 'package:graphics':
##
##     pairs
```

```
plot(NA,NA,xlim=c(-2,2),ylim=c(-2,2),xlab="Uncertainty in the agent-brain size effect",
     ,ylab="Uncertainty in the agent-body size effect")
for(i in 1:(dim(d_sub)[1])){
  c<-diag(3)
  c[1,2]<-c[2,1]<-d_sub$v.body.corr[i]
  c[1,3]<-c[3,1]<-d_sub$v.brain.corr[i]
  c[2,3]<-c[3,2]<-d_sub$brain.body.corr[i]
  estimates<-MoM_estimators(c)
  uncertainty_info<-MC.se(c,n=d_sub$sample.size[i])
  lines(ellipse(uncertainty_info$vcv,centre=estimates),col=i)
}
```

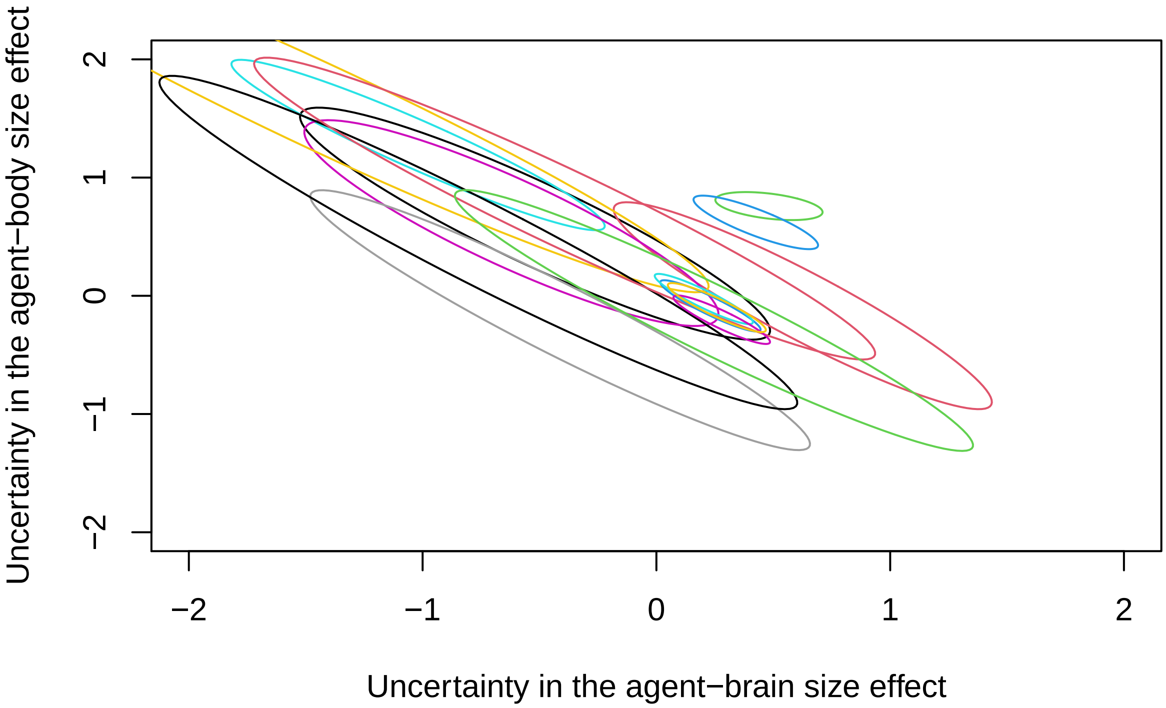

A few estimates have reasonable precision, but even after subsetting for the most powerful of the available studies, most have very large uncertainty. The horizontal and vertical spans of these covariance ellipses are approximately representative of the 95% confidence intervals of the estimates. Note the very strong sampling error covariance between the agent-brain size and agent-body size effects in each study.

The relationship between the 'reversed' multiple regression analysis and direct effect estimates that account for the basic model of reciprocal correlated evolution

Above, we describe how the multiple regression analysis used by Rogel et al (2019) does not recover the partial effects of selective agents on traits, as they are usually understood. However, these quantities do coincide closely (but not perfect) with the estimates of direct effects that account for the basic model of reciprocal correlated evolution:

```
par(mfrow=c(1,2))
plot(d$partial.brain,d$effect_abr,xlim=c(-3,3),ylim=c(-3,3))
abline(0,1,col="red")
plot(d$partial.body,d$effect_abo,xlim=c(-3,3),ylim=c(-3,3))
abline(0,1,col="red")
```

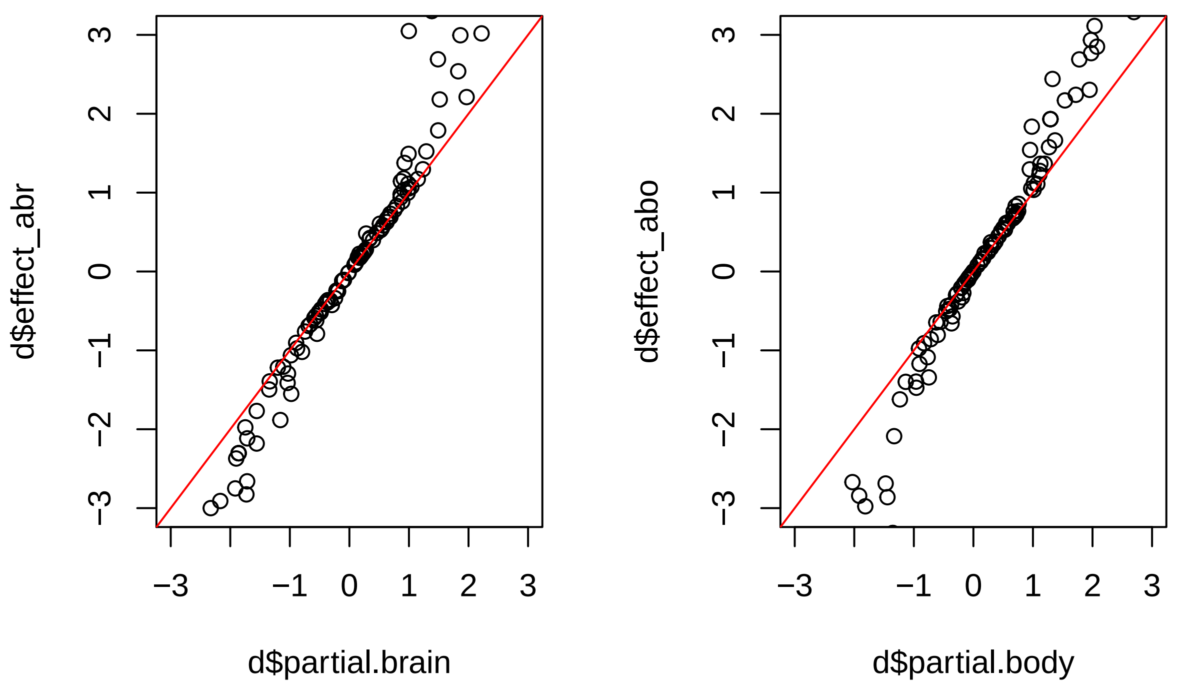

This coincidence is not general. It will only hold under variance standardisation of all variables (though this suggests that some transformation could be employed such that the “reversed” multiple regression could be employed as a rough estimator of the direct effects). For example, the numerical example in which we first applied the esimators from equation 4a/b had non-unit variances:

```
cov(cbind(agent,body,brain))
```

```
##           agent      body     brain
## agent 0.9654711 0.8725536 0.5291656
## body  0.8725536 0.9744987 0.5564412
## brain 0.5291656 0.5564412 0.9912418
```

In this case, the ‘reversed’ multiple regression coefficients do not coincide with the direct effect estimates:

```
MoM_estimators(cov(cbind(agent,body,brain)))
```

```
## agent_brain_effect  agent_body_effect
##          0.1762121          0.8841074
```

```
summary(lm(agent~brain+body))$coefficients
```

```
##                 Estimate  Std. Error     t value     Pr(>|t|)
## (Intercept) 0.0006611959 0.006049047   0.1093058 9.129643e-01
## brain       0.0459312344 0.007369417   6.2326825 4.961764e-10
## body        0.8691603003 0.007432455 116.9412173 0.000000e+00
```

A Bayesian model to estimate direct effects in the presence of reciprocal indirect effects

Developing the MoM estimators was useful as a demonstration that direct effects are estimable from observational data in the presence of a simple model where both traits can evolve due to indirect and direct causes. However, further elaboration of models allowing for this basic phenomenon might be constrained by the MoM approach. As we note in the article, it seems plausible that a Bayesian approach is likely to prove more ammenable to extension to more complicated situations. We do not make any such extensions here, but we do show how the basic principles might be incorporated into a Bayesian analysis.

This section includes a one-off simulation example. We have verified that it generally behaves as expected, but in case some weird sampling error strikes in any one compilation such that estimated parameters could greatly differ from simulated parameters, we have checked that using this random number seed gives sensible results (delete this chunk if you want to play around with multiple independent runs, or to use it to make a simualtion with many replicates of any given scenario):

```
set.seed(99)
```

In this section we'll use the general Bayesian tool, `jags`, and we'll need to simulate data from a multivariate normal distribution:

```
library(rjags)
```

```
## Loading required package: coda
```

```
## Linked to JAGS 4.3.0
```

```
## Loaded modules: basemod,bugs
```

```
library(mvtnorm)
```

This model encodes the basic logic that we used in deriving the MoM estimators of direct effects:

```
m<-"model{

  ### Priors

  # SDs and correlation of brain and body size
  # before effect of the selective agent
  sigma_br~dunif(0.001,10)
  sigma_bo~dunif(0.001,10)
  rho~dunif(-0.99,0.99)

  # the above define the precision matrix
  Sigma[1,1]<-sigma_br^2
  Sigma[2,2]<-sigma_bo^2
  Sigma[1,2]<-rho*sigma_br*sigma_bo
  Sigma[2,1]<-rho*sigma_br*sigma_bo
  Tau[1:2,1:2]<-inverse(Sigma[1:2,1:2])

  # the SDs and correlation also define
  # the regressions of each trait
```

```
  # on the other, which can be organised this
  # way for use in the part of the bivariate
  # response model that accounts for direct
  # and indirect diversification according to
  # the selective agent
  allobeta[1,1]<-1
  allobeta[1,2]<-Sigma[1,2]/Sigma[2,2]
  allobeta[2,1]<-Sigma[1,2]/Sigma[1,1]
  allobeta[2,2]<-1

  # trait means
  mu[1]~dnorm(0,0.001)
  mu[2]~dnorm(0,0.001)

  # regressions defining the direct effects of
  # the agent on brain and body size, arranged
  # in a column vector
  beta[1,1]~dnorm(0,0.001)
  beta[2,1]~dnorm(0,0.001)

  # bivariate model with correlated responses to
  # diversifying selection, and residual variance
  # and covariance, defined by the allometry that would
  # exist over and above whatever covariance of traits
  # is imposed by the focal selective agent
  for(i in 1:n){
    z[i,1:2]~dmnorm(mu[1:2]
                + allobeta[1:2,1:2]%*%(beta[1:2,1]*a[i]),
             Tau[1:2,1:2])
  }
}"
writeLines(m,con=file("./jagsMod.jags"))
```

Here, we simulate data where the selective agent drives diversification in both body size and the focal trait (brain size), and where both have direct responses, as well as indirect evolutionary responses to resulting from direct selection on the other trait.

```
# number of taxa in a brain-body evolutionary allometry study
# making a plausible but large number for the purpose of proof-of-principle
n<-200

# variance and covariance of brain and body that would
# occur (either through immediate correlated responses
# to selection or longer-term processes like correlations
# in selective optima)
cov.mat<-matrix(c(3,1.5,1.5,2),2,2)

# start constructing a data frame; first make up records
# of brain and body size before the selective agent becomes
# relevant
d<-rmvnorm(n,c(3,2), cov.mat)
d<-data.frame(body0=d[,1],brain0=d[,2])
```

```
# here is a selective agent
d$agent<-rnorm(n,0,1)

# direct effects of the selective agent on brain and body size
br.coef <- -0.25
bo.coef <- 0.5

# selective agent acts on body size; direct and indirect components
d$body<- d$body0 + bo.coef*d$agent + cov.mat[1,2]/cov.mat[2,2] * br.coef*d$agent

# same for evolution of brain size
d$brain <- d$brain0 + br.coef*d$agent  + cov.mat[1,2]/cov.mat[1,1] * bo.coef*d$agent
```

To run the model, we package the relevant variables into a list, and initialise a model to be sampled based on the code above:

```
# the information that would naturally be available
dat<-list(
  n=n
 ,z=d[,c("brain","body")]
 ,a=d$agent
)

m1<-jags.model(file="./jagsMod.jags",
  data=dat)
```

```
## Compiling model graph
##    Resolving undeclared variables
##    Allocating nodes
## Graph information:
##    Observed stochastic nodes: 200
##    Unobserved stochastic nodes: 7
##    Total graph size: 1025
##
## Initializing model
```

```
s1<-jags.samples(model=m1,
  variable.names=c("sigma_br","sigma_bo","rho","mu","beta"),
  n.iter=5000,thin=5)
```

We'll jump right to the estimates of the direct effects of the agent on brain size and body size:

```
apply(s1$beta[,1,,1],1,mean)
```

```
## [1] -0.2261848  0.4719018
```

These are reasonable reflections of the direct effects we simulated (see above, they were -0.25 for the effect of the agent on brain size, and +0.50 for the effect of the agent on body size). They correspond even more closely (as expected) to the estimates obtained by MoM from the same data

```r
# use the function for the MoM estimators from way above:
MoM_estimators(cov(d[,c("agent","body","brain")]))
```

```
## agent_brain_effect  agent_body_effect
##         -0.2232552          0.4677421
```
